# Saponins Derived from Chinese Medicinal Materials in Hyperuricemia and Gout: Therapeutic Mechanisms and Emerging Perspectives

**DOI:** 10.3390/molecules31183338

**Published:** 2026-09-20

**Authors:** Zifan Wang, Ying Chen, Tiantian Li, Yu Hu, Lingjun Li

**Affiliations:** School of Pharmacy, Shandong University of Traditional Chinese Medicine, Jinan 250355, China

**Keywords:** saponins, Chinese medicinal materials, hyperuricemia, gout, uric acid metabolism, gut microbiota, inflammatory signaling pathways

## Abstract

Hyperuricemia (HUA) is a metabolic disorder caused by dysregulated uric acid metabolism and constitutes the principal biochemical basis of gout. Persistent elevation of serum uric acid above the saturation threshold promotes monosodium urate crystal deposition in joints, soft tissues, and the kidneys, triggering inflammation and tissue injury. The rising global prevalence of HUA and gout, together with an evident trend toward younger onset, has imposed an increasing public health burden. Traditional Chinese Medicine has been widely used in their management because of its holistic therapeutic approach and favorable safety profile. Among the bioactive constituents derived from Chinese medicinal materials, saponins have attracted particular attention for their multi-target pharmacological actions. Saponin compounds derived from Chinese medicinal materials exert anti-hyperuricemic and anti-gout effects by modulating purine-metabolizing enzymes, regulating urate transporters, restoring gut microbiota homeostasis, and suppressing inflammatory signaling pathways. This review summarizes the pathogenesis of HUA and gout and synthesizes current evidence on the therapeutic mechanisms of saponins derived from Chinese medicinal materials, providing a theoretical basis for the development of novel therapeutic strategies.

## 1. Introduction

Hyperuricemia (HUA) is a metabolic disorder characterized by abnormally elevated serum uric acid (SUA) levels resulting from disturbances in uric acid metabolism [1]. When SUA concentrations remain above the saturation threshold of 420 μmol/L for a prolonged period [2], monosodium urate (MSU) precipitates as needle-like crystals and progressively deposits in the joints, soft tissues, and kidneys. These deposits trigger local inflammatory responses and tissue damage, ultimately contributing to the development of gout [3]. Thus, HUA constitutes the biochemical basis of gout, whereas gouty arthritis (GA), driven by MSU crystal deposition, represents its predominant clinical manifestation [4].

According to the Global Burden of Disease study, the age-standardized prevalence of gout worldwide increased by 22.5% between 1990 and 2020, with the number of affected individuals reaching 55.8 million in 2020 and projected to rise further to 95.8 million by 2050 [5]. The prevalence of gout shows marked geographic, sex-related, and age-related variation, being higher in high-income regions than in low- and middle-income regions, substantially higher in men than in women, and increasing progressively with age [6]. In China, rising living standards and accompanying shifts in dietary patterns have contributed to a rapid increase in the prevalence of both HUA and gout, which have reached 17.7% and 3.2%, respectively, among adults. Of particular concern, the age-standardized prevalence of gout among young adults in China is 262.73 per 100,000, markedly exceeding the corresponding global average of 170.68 per 100,000 [7]. Collectively, these epidemiological trends indicate a clear shift toward earlier gout onset and highlight the increasing challenges associated with its prevention and control.

Current Western medical management of HUA and gout primarily focuses on lowering SUA levels with xanthine oxidase inhibitors, such as allopurinol and febuxostat, and uricosuric agents, such as benzbromarone. During acute gout attacks, colchicine, glucocorticoids, and nonsteroidal anti-inflammatory drugs, including etoricoxib, are commonly used to control inflammation and relieve symptoms [8]. However, long-term or repeated administration of these agents is associated with a range of adverse reactions, including cutaneous hypersensitivity, increased cardiovascular risk, hepatic and renal injury [9], and gastrointestinal damage [10], which may limit their prolonged clinical use.

From the perspective of Traditional Chinese Medicine (TCM), HUA is primarily associated with the pathological concepts of “latent pathogenic factors” and “blood turbidity,” whereas gouty arthritis is generally classified within the categories of “bi syndrome” and “joint obstruction” [11]. On the basis of long-term clinical practice, TCM has developed extensive experience in syndrome differentiation and treatment and may offer distinct advantages in controlling disease progression, reducing recurrence, and minimizing treatment-related adverse effects. In recent years, saponins derived from Chinese medicinal materials (CMMs) have attracted increasing attention because of their multi-target pharmacological effects; they exert anti-hyperuricemic and anti-gout effects through multiple mechanisms, including modulation of the activities of enzymes and urate transporters involved in uric acid metabolism, as well as against HUA and gout. Available studies indicate that these compounds maintain gut microbiota homeostasis and regulate inflammation-related signaling pathways.

Accordingly, this review summarizes the current understanding of the pathogenesis of HUA and gout and systematically discusses the therapeutic mechanisms of saponins derived from CMMs in these disorders, with the aim of providing a theoretical basis and new perspectives for the development of novel therapeutic agents and for improving the clinical prevention and management of HUA and gout.

## 2. Literature Search Strategy

A literature search was conducted using PubMed and the China National Knowledge Infrastructure (CNKI) databases to identify relevant studies regarding the pharmacological effects and mechanisms of saponins derived from Chinese medicinal materials in hyperuricemia and gout. The literature search included relevant publications available up to August 2026. The main search terms included “saponin”, “hyperuricemia”, “gout”, and “uric acid”, with additional related terms such as “urate transporter”, “inflammation”, and “gut microbiota” used when necessary.

Relevant publications were reviewed based on their scientific relevance and contribution to the topic, and representative studies were selected for discussion. Studies focusing solely on phytochemical characterization without pharmacological evaluation were excluded. In addition, studies based only on network pharmacology prediction without experimental validation, or those reporting urate-lowering activity without further investigation of the underlying mechanisms, were not considered. The literature discussed in this review mainly includes preclinical studies, including in vitro cellular experiments and in vivo animal models.

## 3. Pathogenesis of HUA and Gout

The onset and progression of HUA and gout are driven by multiple interconnected pathological processes, including disturbances in purine metabolism, dysregulation of urate transport, gut microbiota imbalance, and aberrant activation of inflammatory signaling pathways. Rather than acting independently, these mechanisms interact and reinforce one another, collectively contributing to disease initiation and progression.

### 3.1. Enzymes Involved in Purine Metabolism—Increased Uric Acid Production

Uric acid is the end product of purine metabolism and is generated from purine nucleotides through a series of enzymatic degradation and oxidation reactions. Endogenous purine nucleotides are primarily derived from two pathways: de novo synthesis and the salvage pathway [12]. Purine nucleotide synthesis is tightly regulated by precursor availability and the activities of key metabolic enzymes. Accordingly, aberrant expression or dysregulation of rate-limiting enzymes can disrupt purine nucleotide metabolism, ultimately leading to excessive uric acid production.

Phosphoribose pyrophosphate synthase (PRPS) is a key rate-limiting enzyme in the de novo biosynthesis of purine nucleotides and catalyzes the formation of phosphoribosyl pyrophosphate (PRPP), an essential precursor for purine synthesis [13]. Increased PRPS transcription or abnormally elevated enzymatic activity results in excessive PRPP production, which in turn enhances the activity of downstream enzymes [14], including phosphoribosyl pyrophosphate acyltransferase (PRPPAT). This promotes excessive purine nucleotide synthesis and consequently increases uric acid production.

Adenosine deaminase (ADA) catalyzes the conversion of adenosine monophosphate to hypoxanthine monophosphate [15]. Abnormally elevated ADA activity accelerates this conversion [16], thereby increasing the availability of substrates for uric acid synthesis and ultimately contributing to excessive uric acid production.

Xanthine oxidoreductase (XOR) is a key rate-limiting enzyme in uric acid biosynthesis and exists in two interconvertible forms, xanthine oxidase (XOD) and xanthine dehydrogenase (XDH) [17]. XOR catalyzes the sequential oxidation of hypoxanthine to xanthine and subsequently to uric acid. Accordingly, upregulated XOR expression or abnormally increased enzymatic activity can directly enhance uric acid production. In addition to its role in purine catabolism, XOD generates reactive oxygen species (ROS) during uric acid formation [18], thereby promoting oxidative stress and aggravating tissue damage.

**Figure 1:**
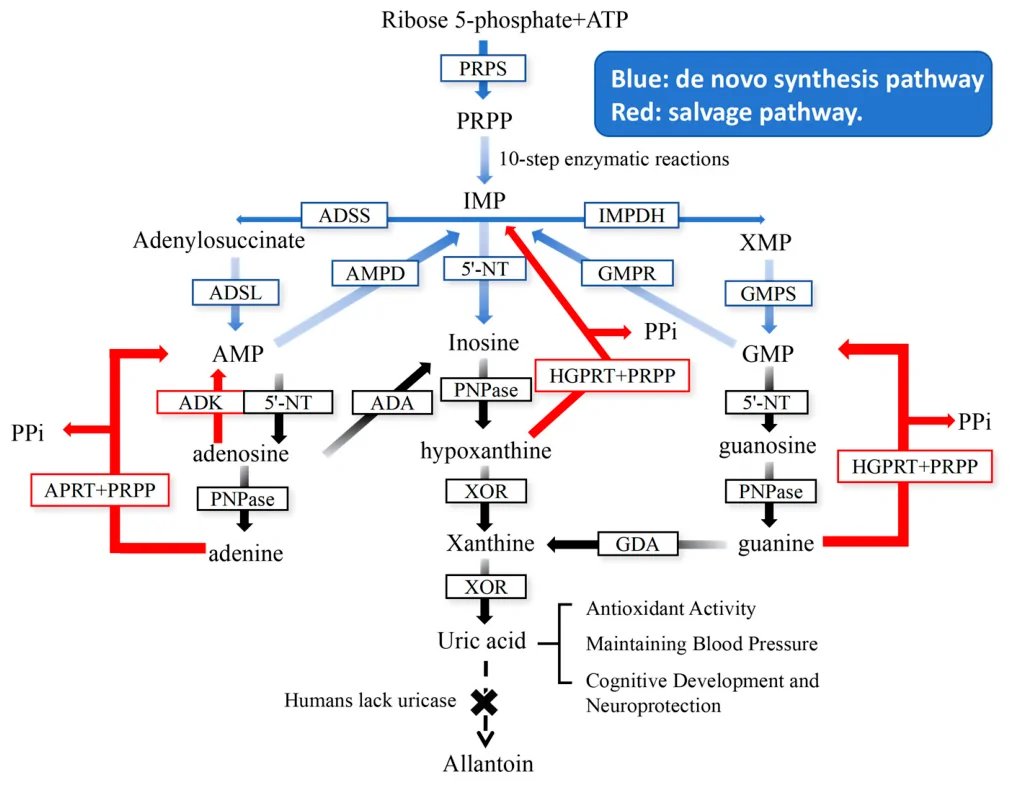
Uric acid synthesis.

Hypoxanthine–guanine phosphoribosyltransferase (HGPRT) is a key enzyme in the purine salvage pathway [19]. It catalyzes the utilization of free hypoxanthine and guanine, together with PRPP, to generate hypoxanthine and guanine nucleotides, thereby reducing the pool of purine bases available for subsequent uric acid production. When HGPRT activity is reduced or absent, impairment of the salvage pathway leads to the accumulation of hypoxanthine and guanine. These accumulated purine bases are subsequently directed toward degradation, resulting in excessive uric acid production The major enzymatic pathways involved in purine metabolism and uric acid synthesis are summarized in Figure 1. Despite its pathogenic effects when excessively accumulated, urate also exerts several potentially beneficial physiological effects. As an important antioxidant in human biological fluids, urate scavenges free radicals and chelates metal ions, thereby limiting oxidative damage. It may also contribute to the maintenance of blood pressure under low-salt conditions and exert neuroprotective effects by attenuating oxidative stress and glutamate-induced neuronal toxicity [20,21].

### 3.2. Urate Transporters—Reduced Urate Excretion

The kidneys play a central role in maintaining uric acid homeostasis, accounting for approximately 70% of total urate excretion [22]. Renal urate handling involves coordinated reabsorption and secretion, processes that depend on the activity of multiple urate transporters encoded by different genes and expressed on the apical and basolateral membranes of renal tubular epithelial cells. These transporters constitute the molecular basis of renal urate transport; consequently, alterations in their expression or functional impairment can disrupt renal urate handling and represent important determinants of SUA levels [23].

Renal urate reabsorption is mediated primarily by urate transporter 1 (URAT1, encoded by the *SLC22A12* gene) [24] and organic anion transporter 4 (OAT4, encoded by the *SLC22A11* gene) [25], both of which are located on the apical membrane of renal tubular epithelial cells. Through exchange with intracellular organic anions, URAT1 and OAT4, respectively, utilize monocarboxylates, such as lactate, and dicarboxylates, such as α-ketoglutarate, to mediate urate uptake from the tubular lumen into epithelial cells. Glucose transporter 9b (GLUT9b, encoded by the *SLC2A9* gene), also expressed on the apical membrane, further contributes to luminal urate uptake through facilitated diffusion [26]. The intracellular pool of exchangeable organic anions is maintained by coordinated transmembrane transport: sodium-coupled monocarboxylate transporter 1 (SMCT1, encoded by the *SLC5A8* gene) on the apical membrane actively mediates monocarboxylate uptake [27], whereas sodium-dependent dicarboxylate transporter 3 (NaDC3, encoded by the *SLC13A3* gene) on the basolateral membrane transports dicarboxylates from the circulation into tubular epithelial cells [28]. Following apical uptake, intracellular urate is transported across the basolateral membrane into the circulation by glucose transporter 9a (GLUT9a, encoded by the *SLC2A9* gene) [26].

Renal urate secretion proceeds in the opposite direction. Organic anion transporter 1 (OAT1, encoded by the *SLC22A6* gene) and organic anion transporter 3 (OAT3, encoded by the *SLC22A8* gene) [29], located on the basolateral membrane, mediate urate uptake from the blood into tubular epithelial cells through exchange with intracellular dicarboxylates. Urate is subsequently exported across the apical membrane into the tubular lumen by ATP-binding cassette subfamily G member 2 (ABCG2, encoded by the *ABCG2* gene) and ATP-binding cassette subfamily C member 4 (ABCC4, encoded by the *ABCC4* gene), both of which utilize energy derived from ATP hydrolysis [30]. In parallel, sodium-dependent phosphate transporter 1 (NPT1, encoded by the *SLC17A1* gene) and sodium-dependent phosphate transporter 4 (NPT4, encoded by the *SLC17A3* gene) facilitate urate secretion into the urine through voltage-dependent transport mechanisms [31].

**Figure 2:**
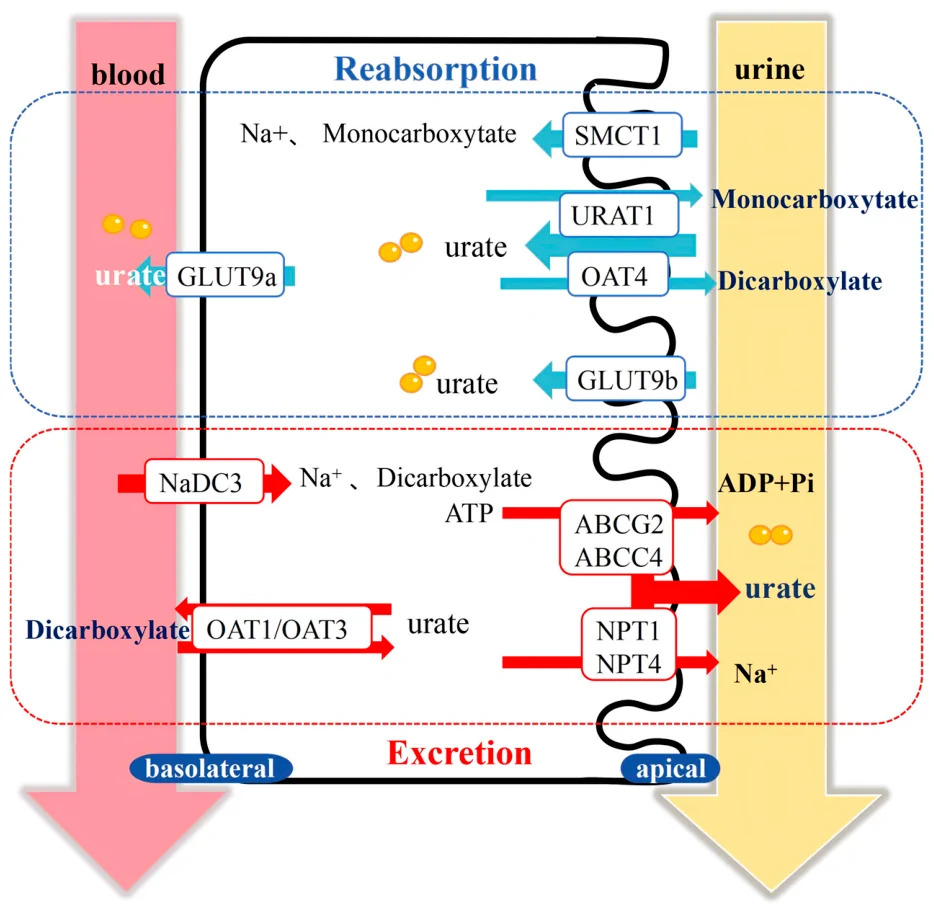
Urate transporters in renal tubular epithelial cells. Blue arrows and outlines indicate urate reabsorption-related transport pathways, whereas red arrows and outlines indicate urate excretion-related transport pathways.

Given the central role of these transport systems in renal urate handling, targeted modulation of the expression and function of renal urate transporters has emerged as an important strategy for reducing SUA levels. The major renal urate transporters involved in urate reabsorption and secretion are summarized in Figure 2.

### 3.3. Gut Microbiome Dysbiosis

The intestine plays dual roles in exogenous purine absorption and urate transport. It is a major site for the absorption of dietary purines, which account for approximately 20% of the body’s total purine content [32], while also serving as the principal route of extrarenal urate excretion, responsible for approximately 30% of total uric acid elimination. At the molecular level, transporters located on intestinal epithelial cells coordinate this bidirectional urate flux. ATP-binding cassette transporters, including ABCG2 and ABCC4, mediate the efflux of uric acid across the apical membrane into the intestinal lumen, whereas GLUT9 on the basolateral membrane participates in urate reabsorption [33]. Together, these transporters constitute the molecular basis of intestinal urate transport. However, efficient intestinal urate excretion depends not only on transporter function but also on the maintenance of gut microbiota homeostasis and intestinal barrier integrity.

The gut microbiota can partially compensate for the host’s lack of uricase activity. Several bacterial species, including *Bacillus subtilis*, *Proteus mirabilis*, and *Escherichia coli*, harbor uricase [34], enabling the conversion of uric acid into allantoin and urea for further degradation. Other microorganisms, such as *Clostridium perfringens*, *Escherichia coli I-11*, and *MS 200-1*, are capable of utilizing purine bases [35], thereby reducing the availability of substrates for uric acid synthesis. In addition, bacteria harboring the uric acid gene cluster can metabolize uric acid into downstream products, including xanthine, lactic acid, and short-chain fatty acids (SCFAs) [36]. Collectively, these microbial metabolic activities contribute to intestinal uric acid disposal and purine homeostasis.

As HUA progresses to gout, however, the richness and diversity of the gut microbiota progressively decline. The *Firmicutes*/*Bacteroidetes* (F/B) ratio, an important indicator of intestinal microbial homeostasis, shows a compensatory increase during the asymptomatic HUA stage but decreases significantly after the onset of gout [37]. This shift is accompanied by a reduction in beneficial bacteria with uric acid-degrading capacity, including *Lactobacillus* and *Ruminococcus* [38], together with an increased abundance of genera such as *Escherichia* and *Shigella*, which secrete or induce XOR production and thereby promote uric acid formation [39]. Pro-inflammatory genera, including *Prevotella* and *Fusobacterium*, are likewise enriched [40]. Their expansion further promotes intestinal inflammation and downregulates Zonula occludens-1 (ZO-1), occludin, and other tight junction proteins, resulting in disruption of intestinal barrier integrity [41] and subsequent translocation of lipopolysaccharide (LPS) into the circulation. Once in the bloodstream, LPS enhances XOD activity, thereby increasing ROS generation and uric acid production [42], while simultaneously activating inflammatory signaling pathways and promoting the release of inflammatory mediators [43]. These interrelated processes establish a self-reinforcing cycle of elevated uric acid–gut microbiota dysbiosis–aggravated inflammation, which may further drive the progression from HUA to gout.

Metabolites derived from the gut microbiota serve as important mediators linking intestinal uric acid metabolism to the local intestinal microenvironment [38]. Among these metabolites, SCFAs exert multiple regulatory effects on uric acid homeostasis. SCFAs reduce uric acid and ROS production by inhibiting XDH expression and promoting its conversion to XOD [44], while also enhancing intestinal urate excretion through upregulation of ABCG2 expression [45]. Beyond their direct effects on uric acid metabolism, SCFAs help reshape the gut microbial ecosystem by enriching SCFAs-producing beneficial bacteria and restraining the excessive proliferation of pathogenic taxa [46]. They also contribute to the restoration of intestinal barrier integrity by increasing the expression of tight junction proteins and hypoxia-inducible factors in intestinal epithelial cells [47]. These effects collectively limit the translocation of LPS into the circulation and thereby interrupt the self-reinforcing cycle between disturbed uric acid metabolism and inflammation. In contrast, potentially harmful intestinal metabolites, including the uremic toxins trimethylamine-N-oxide and indophenol sulfate, can aggravate intestinal injury and renal inflammation, further amplifying the pathological damage associated with HUA [48].

**Figure 3:**
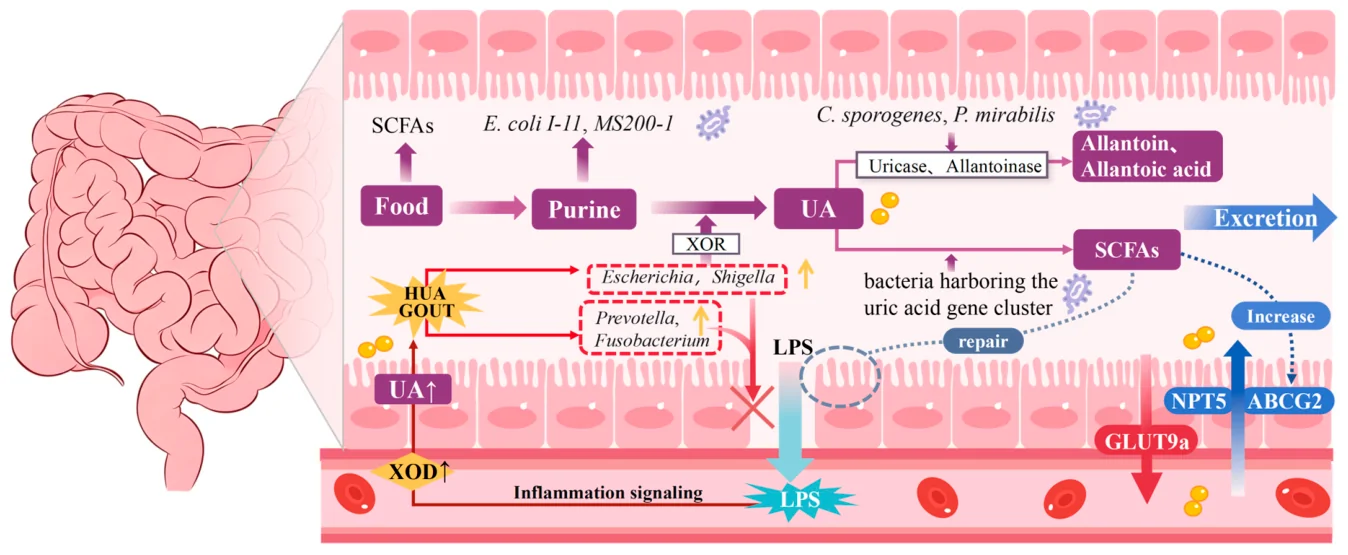
The gut microbiome and uric acid metabolism. Purple arrows indicate microbial purine and uric acid metabolic pathways; red arrows indicate processes associated with urate accumulation and inflammation; and blue arrows indicate protective processes, including intestinal barrier repair and enhanced urate excretion.

Taken together, beneficial gut bacteria and their metabolites constitute an important regulatory component of intestinal uric acid metabolism. Targeted modulation of the gut microbiota and its metabolic products may therefore represent a promising therapeutic strategy for HUA and gout. The interactions among the gut microbiota, uric acid metabolism, intestinal barrier integrity, and intestinal urate excretion are summarized in Figure 3.

### 3.4. Abnormal Activation of Inflammatory Signaling Pathways

The inflammatory response constitutes the central pathological basis underlying acute gout attacks and subsequent tissue injury. Acting as endogenous danger signals, MSU crystals are recognized by pattern-recognition receptors on innate immune cells, thereby activating multiple inflammation-related signaling pathways and initiating an amplified inflammatory cascade [49]. These interconnected pathways collectively promote the production and release of pro-inflammatory mediators, facilitate the recruitment and activation of inflammatory cells, and ultimately contribute to local tissue damage.

#### 3.4.1. TLRs/MyD88/NF-κB Signaling Pathway

MSU crystals are recognized by innate immune cells through Toll-like receptor 2 (TLR2) and Toll-like receptor 4 (TLR4) [50], initiating a myeloid differentiation primary response 88 (MyD88)-dependent signaling and the sequential activation of interleukin-1 receptor-associated kinase 1 (IRAK1), TNF receptor-associated factor 6 (TRAF6), and the transforming growth factor-β-activated kinase 1 (TAK1) complex. This signaling cascade subsequently activates the IκB kinase (IKK) complex [51], promoting IκBα phosphorylation and degradation and thereby releasing nuclear factor-κB (NF-κB) heterodimers, predominantly p65/p50, which subsequently translocate into the nucleus. Following nuclear translocation, NF-κB promotes the transcription of pro-inflammatory genes, including those encoding tumor necrosis factor-α (TNF-α), interleukin-6 (IL-6), interleukin-1β precursor (pro-IL-1β), and interleukin-18 precursor (pro-IL-18), and upregulates NLR family pyrin domain-containing 3 (NLRP3) expression [52], thereby providing the priming signal for subsequent NLRP3 inflammasome assembly. Collectively, activation of this signaling cascade contributes to the rapid amplification of MSU crystal-induced inflammation during acute gout flares.

#### 3.4.2. NLRP3 Inflammasome Signaling Pathway

The NLRP3 inflammasome represents a central signaling platform in the inflammatory response underlying gout and is activated through two sequential stages: priming and activation. During the priming stage, activation of the NF-κB pathway or other upstream signaling pathways increases the transcription and expression of NLRP3 and inflammasome-related precursor molecules. In the subsequent activation stage, exposure of immune cells to pathologically elevated concentrations of uric acid [53] or phagocytosis of MSU crystals triggers three major intracellular danger signals: K^+^ efflux, increased mitochondrial reactive oxygen species (ROS) production, and lysosomal rupture. These events promote conformational changes and oligomerization of NLRP3 [54], followed by recruitment of apoptosis-associated speck-like protein containing a caspase recruitment domain (ASC). ASC, in turn, recruits pro-caspase-1, promoting NLRP3 inflammasome assembly and the subsequent cleavage and activation of pro-caspase-1.

Activated caspase-1 then specifically cleaves pro-IL-1β and pro-IL-18 into the mature pro-inflammatory cytokines interleukin-1β (IL-1β) and interleukin-18 (IL-18), respectively. These cytokines act as direct effector molecules in triggering acute gout flares. In parallel, activated caspase-1 cleaves gasdermin D (GSDMD), relieving its autoinhibitory state and inducing pyroptosis. The subsequent release of intracellular inflammatory mediators [55] further amplifies the inflammatory cascade, thereby exacerbating the acute inflammatory response in gout.

#### 3.4.3. MAPK Signaling Pathway

The mitogen-activated protein kinase (MAPK) signaling pathway comprises three canonical branches: extracellular signal-regulated kinase 1/2 (ERK1/2), c-Jun N-terminal kinase (JNK), and p38 MAPK. Following recognition of MSU crystals by TLR2/TLR4 on innate immune cells, TAK1 is activated through MyD88-dependent signaling, initiating a three-tiered MAPK cascade. This process leads to the phosphorylation of members of the mitogen-activated protein kinase kinase (MKK) family, including MEK1/2, MKK3/6, and MKK4/7, which subsequently activate ERK1/2, p38 MAPK, and JNK, respectively [56].

Although these three MAPK branches are closely interconnected, their regulatory roles in gout-associated inflammation differ. p38 MAPK primarily drives the transcription of pro-inflammatory cytokine genes, including those encoding TNF-α and IL-1β, and can prolong their inflammatory effects by enhancing mRNA stability. JNK signaling is more closely involved in the expression of matrix metalloproteinases (MMPs) and apoptosis and is therefore closely associated with joint tissue destruction. ERK1/2 plays a predominant role in early inflammatory signal amplification; it can phosphorylate and activate the IκB kinase (IKK) complex, promote IκBα degradation, and thereby indirectly enhance NF-κB transcriptional activity [57].

Beyond regulating cytokine production, MAPK activation also promotes the expression of intercellular adhesion molecule 1 (ICAM-1), vascular cell adhesion molecule 1 (VCAM-1), and C-X-C motif chemokine ligand 1 (CXCL1) [58], thereby facilitating inflammatory cell adhesion, recruitment, and infiltration into affected joints. MAPK signaling has also been implicated in neutrophil extracellular trap (NET) formation [59], which may further amplify the local inflammatory response in gout.

#### 3.4.4. PI3K/AKT Signaling Pathway

MSU crystals promote the clustering of immunoreceptor tyrosine-based activation motif (ITAM)-coupled receptors, leading to the sequential activation of Src family kinases (SFKs) and spleen tyrosine kinase (Syk) [60]. Activated Syk subsequently phosphorylates and activates phosphoinositide 3-kinase (PI3K), relieving the inhibitory effect of its regulatory p85 subunit on the catalytic p110 subunit. PI3K then catalyzes the conversion of phosphatidylinositol 4,5-bisphosphate (PIP2) to phosphatidylinositol 3,4,5-trisphosphate (PIP3), thereby promoting the recruitment of the serine/threonine kinase AKT to the plasma membrane. At the plasma membrane, AKT is phosphorylated and activated through the coordinated actions of 3-phosphoinositide-dependent protein kinase-1 (PDK1) and mechanistic target of rapamycin complex 2 (mTORC2). Activated AKT subsequently activates mechanistic target of rapamycin complex 1 (mTORC1), a key negative regulator of autophagy. By suppressing autophagy initiation, mTORC1 impairs the clearance of damaged mitochondria and MSU crystals, thereby exacerbating cellular injury and sustaining the inflammatory response [61].

However, the effects of MSU crystals on PI3K/AKT signaling appear to be context-dependent. In human chondrocytes derived from patients with osteoarthritis, MSU crystals instead inhibit activation of the PI3K/AKT pathway [62]. This relieves mTORC1-mediated inhibition of autophagy, induces autophagic cell death in chondrocytes, and consequently contributes to articular cartilage damage.

**Figure 4:**
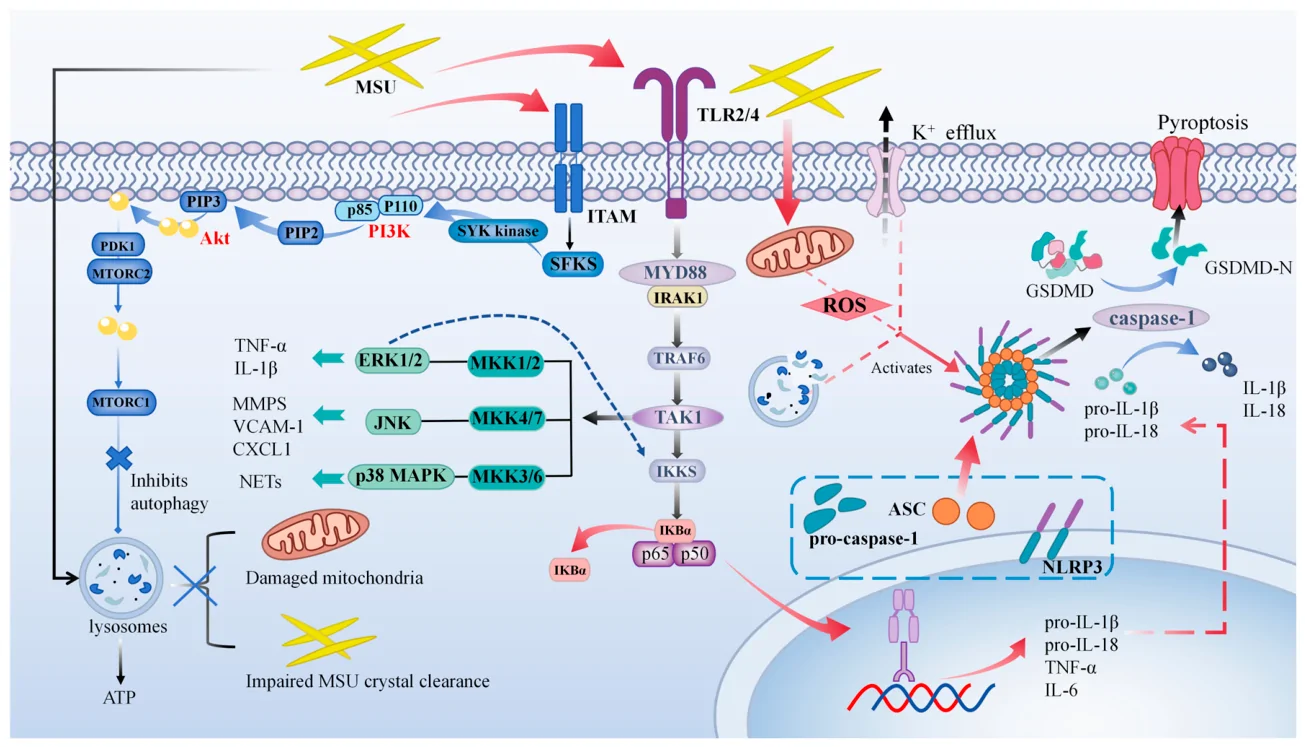
Regulatory mechanisms of multiple inflammatory signaling pathways in gout.

The inflammatory signaling pathways described above do not function in isolation. Rather, they form an interconnected signaling network in which shared upstream stimuli and multiple cross-regulatory nodes enable reciprocal activation and amplification of inflammatory responses. Such extensive pathway crosstalk may limit the effectiveness of interventions directed at a single signaling axis. In this context, saponin compounds derived from CMMs, which can coordinately modulate multiple inflammatory pathways through multi-target mechanisms, may offer broader therapeutic advantages in the anti-inflammatory management of gout. The interactions among these inflammatory signaling pathways and their regulatory mechanisms in gout are summarized in Figure 4.

## 4. Experimental Models for Evaluating the Therapeutic Effects of Saponins Against HUA and Gout

Various animal models have been employed to evaluate the hypouricemic activity of saponin compounds, as different modeling strategies reproduce distinct pathological features of hyperuricemia. Unlike humans, rodents possess uricase, which converts uric acid into allantoin and results in substantially lower basal SUA levels. Accordingly, experimental hyperuricemia is commonly established by manipulating uric acid production or degradation to generate a pathological state more comparable to that in humans [63]. Among these approaches, the potassium oxonate (PO)-induced model is one of the most widely used for pharmacological evaluation. By inhibiting uricase activity and reducing the conversion of uric acid to allantoin, PO rapidly elevates SUA levels and produces a reproducible hyperuricemic phenotype, making this model particularly suitable for preliminary screening of urate-lowering agents [64]. However, because PO alone predominantly reflects impaired uric acid degradation, it is often combined with additional inducers, such as hypoxanthine, high-purine diets, or fructose, to further enhance uric acid production or introduce metabolic disturbances, thereby producing a more sustained and stable hyperuricemic state. By contrast, high-purine diet-associated models, including yeast extract-induced hyperuricemia, increase uric acid production primarily through excessive exogenous purine intake and therefore more closely reflect the contribution of dietary factors to HUA development [65]. Such models are particularly useful for evaluating the effects of natural compounds on hyperuricemia associated with excessive purine consumption.

The major animal models used to evaluate the hypouricemic and anti-gout effects of saponin compounds, together with their induction methods, changes in SUA levels, and principal characteristics, are summarized in Table 1.

## 5. Mechanisms of Action of Saponins in the Treatment of HUA and Gout

### 5.1. Regulation of Enzymes Involved in Purine Metabolism

Saponins derived from CMMS can effectively reduce SUA levels by modulating the activity of key enzymes involved in purine metabolism.

*Smilax riparia* A. DC. (Niuweicai), whose roots and rhizomes are used medicinally, is traditionally considered to dispel wind, unblock the meridians, relax the tendons, and relieve pain, and is commonly used for conditions such as rheumatic arthralgia. Total saponins from *S. riparia*, chemically characterized by HPLC-ELSD with 16 identified saponin constituents, exhibited urate-lowering activity in PO-induced HUA mice at 500 mg/kg. Compared with the model group, total saponins alone reduced SUA levels by approximately 19%, whereas allopurinol (5 mg/kg) reduced SUA by approximately 29%. Notably, co-administration of total saponins with allopurinol further decreased SUA levels, resulting in an approximately 50% reduction compared with the model group. Similar effects were observed for serum XOD activity, which was reduced by approximately 23% by total saponins alone and 47% by the combination treatment [74]. These findings suggest that total saponins from *S. riparia* may enhance the urate-lowering efficacy of allopurinol, possibly through complementary inhibition of XOD activity. However, because no formal pharmacological synergy analysis was performed, such as combination index or Bliss independence analysis, the observed interaction should be considered an enhanced combination effect rather than confirmed synergism.

Further studies have identified several steroidal saponins that contribute to the uric acid-lowering activity of total saponins from *S. riparia.* Pallidifloside D, isolated from the total saponins of *S. riparia*, also enhanced the urate-lowering effect of allopurinol in PO-induced HUA mice. Pallidifloside D alone reduced SUA levels by approximately 11%, compared with 29% for allopurinol alone, whereas the combination treatment achieved an approximately 47% reduction. Consistent with its established role as an XOD inhibitor, allopurinol alone reduced XOD activity by approximately 35%, while Pallidifloside D alone showed only a limited inhibitory effect (approximately 2%). Notably, the combination treatment further reduced XOD activity by approximately 41% [75]. These findings suggest that the enhanced urate-lowering effect of the combination treatment may not be solely attributed to XOD inhibition, and that Pallidifloside D may contribute through additional regulatory mechanisms beyond direct XOD inhibition. Subsequent studies demonstrated that Pallidifloside D modulates the mRNA expression levels of purine metabolism-related enzymes, including PRPS, PRPPAT, and HGPRT [76].

Other steroidal saponins, including smilaxchinoside A, smilaxchinoside C, riparoside B, and timosaponin J, also exert uric acid-lowering effects by regulating XOD [77,78], thereby interfering with the terminal oxidative steps of purine metabolism and reducing endogenous uric acid formation. In PO-induced HUA mice, smilaxchinoside A and smilaxchinoside C (10 mg/kg, 7 days) reduced SUA by approximately 22% and 21%, respectively, and decreased XOD activity by approximately 27% and 25%, respectively. However, their urate-lowering effects remained weaker than those of allopurinol at the same dose [77]. At 10 mg/kg, riparoside B and timosaponin J reduced SUA levels from 6.41 ± 0.62 mg/L in the model group to 4.03 ± 0.51 and 5.01 ± 0.47 mg/L, corresponding to reductions of approximately 37% and 22%, respectively. These effects were accompanied by decreased serum XOD activity, with reductions of approximately 22% and 19%, respectively. Compared with allopurinol treatment (3.76 ± 0.37 mg/L), both compounds exhibited weaker but significant urate-lowering activity, suggesting that these steroidal saponins attenuate uric acid production primarily through inhibition of XOD-mediated purine metabolism [78]. Collectively, these individual steroidal saponins act at distinct regulatory nodes involved in purine and uric acid metabolism, providing component-level evidence for the uric acid-lowering activity of total saponins from *S. riparia* and further elucidating the molecular basis of their multi-target action. Although pallidifloside D regulates a broader range of purine metabolism-related enzymes than XOD-focused saponins, available evidence does not support a direct relationship between the number of regulated targets and the magnitude of SUA reduction. Differences in experimental models, dosage, and treatment conditions should also be considered when interpreting these comparisons.

Gypenosides, the total saponins derived from *Gynostemma pentaphyllum* (Thunb.) Makino (Jiaogulan), dose-dependently inhibit XOD and ADA activities in both the serum and liver, while also suppressing hepatic XDH and guanine deaminase (GDA) activities [79]. In lipid emulsion-induced hyperuricemic rats, gypenosides (60 mg/kg) significantly reduced SUA levels, with the high-dose group showing a 32.7% reduction compared with the model group after 8 weeks of treatment. Quantitative analysis of enzyme activities indicated that gypenosides reduced XOD, ADA, GDA, and XDH activities by approximately 18%, 10%, 11%, and 22%, respectively, suggesting simultaneous regulation of multiple enzymatic steps involved in purine degradation [79]. GDA catalyzes the conversion of guanine to xanthine, thereby supplying substrate for subsequent uric acid formation. Through the concurrent inhibition of upstream substrate-generating enzymes and downstream uric acid-producing enzymes, gypenosides interfere with purine-to-uric acid conversion at multiple steps, ultimately reducing uric acid levels.

Both saikosaponin D and total saponins from *Dioscorea nipponica* Makino (Chuanshanlong) significantly inhibit XOD and ADA activities [80,81]. By suppressing these key enzymes involved in purine metabolism, both agents interfere with critical catalytic steps leading to uric acid formation, thereby reducing endogenous uric acid production and lowering SUA levels. In adenine- and ethambutol-induced HUA rats, saikosaponin D administration (100 or 200 mg/kg) reduced SUA levels from 42.29 ± 5.13 mg/L in the model group to 30.48 ± 7.33 and 33.19 ± 5.45 mg/L, corresponding to reductions of approximately 28% and 22%, respectively. These effects were accompanied by decreases in serum XOD activity of approximately 19–22% and ADA activity of approximately 48–62% at the corresponding doses. Collectively, these findings suggest that saikosaponin D contributes to uric acid reduction primarily through inhibition of key enzymes involved in uric acid biosynthesis [80]. Total saponins from *D. nipponica* (60, 600 mg/kg) significantly reduced SUA levels in hyperuricemic mice, with reductions of approximately 29.4% at 60 mg/kg and 43% at 600 mg/kg. At 600 mg/kg, serum XOD and ADA activities were also reduced by approximately 15% and 14%, respectively [81]. In addition to its uric acid-lowering effect, saikosaponin D reduces creatinine and blood urea nitrogen levels and ameliorates HUA-associated renal dysfunction. Notably, the extent of XOD and ADA inhibition did not consistently correspond to the magnitude of SUA reduction among different treatments, suggesting that the urate-lowering effects of these saponins cannot be attributed solely to the inhibition of these two enzymes.

*Apostichopus japonicus*, traditionally used as both a food and medicinal resource, contains saponins with uric acid-lowering activity. In yeast extract powder-induced HUA mice, dietary administration of total saponins from *A. japonicus* with a purity of 91% at 0.05% and 0.1% for 14 days reduced SUA levels from 388 ± 59 μmol/L in the model group to 182 ± 33 and 172 ± 49 μmol/L, corresponding to reductions of 53.1% and 55.6%, respectively. These reductions were accompanied by decreases in hepatic XOD activity of 26.3% and 28.6% and ADA activity of 24.4% and 34.0%, respectively, supporting the involvement of coordinated regulation of purine-metabolizing enzymes in their urate-lowering effects [82]. These coordinated changes support the involvement of XOD and ADA inhibition in the urate-lowering effect of total saponins from *A. japonicus*. By suppressing ADA-mediated substrate generation and the terminal XOD-catalyzed steps of purine metabolism, these saponins may limit endogenous uric acid production and thereby reduce circulating uric acid levels. Notably, total saponins from *A. japonicus* do not adversely affect liver- and kidney-related biochemical parameters, including serum creatinine and blood urea nitrogen, indicating a favorable safety profile. These findings provide a pharmacological basis for the potential use of total saponins from *A. japonicus* in dietary interventions and the development of related products for the prevention and management of HUA and gout.

Flaccidoside II, an oleanane-type triterpene saponin isolated from the rhizomes of *Anemone flaccida* Fr. Schmidt (Ezhangcao), exhibits urate-lowering activity in PO-induced HUA mice, with oral administration at 8, 16, and 32 mg/kg for 7 days reducing SUA by 26.1%, 26.6%, and 30.9%, respectively [83]. At 32 mg/kg, Flaccidoside II reduced hepatic XOD activity by approximately 15%, whereas the lower doses reduced SUA without significantly affecting XOD activity. The inhibitory effect on hepatic XOD at 32 mg/kg was comparable to that of allopurinol (10 mg/kg), although the urate-lowering effect of Flaccidoside II remained weaker than that of allopurinol [83]. Notably, this effect appears to be confined to the pathological state of HUA, as Flaccidoside II does not alter SUA levels in normal mice. This selective pharmacological profile suggests that it may lower uric acid under hyperuricemic conditions without causing excessive urate reduction under physiological conditions.

Collectively, saponin studies show no consistent quantitative link between the number of modulated purine-metabolizing enzymes and SUA reduction. Pallidifloside D modulated PRPS, PRPPAT, and HGPRT, yet when administered alone it lowered SUA by only approximately 11% and inhibited XOD by only about 2% [76]. Gypenosides inhibited four enzymes but reduced SUA by approximately 32.7% [79], whereas the total from saponins *D. nipponica*, mainly affecting XOD and ADA, reduced SUA by approximately 43% [81]. Flaccidoside II lowered SUA by 26.1–30.9% despite being primarily linked to XOD inhibition [83]. Thus, neither enzyme number nor XOD inhibition alone predicts SUA reduction. Because experimental conditions differed, direct comparative studies under identical conditions are needed.

### 5.2. Regulation of Urate Transporters

Saponins exert their anti-hyperuricemic and anti-gout effects by regulating the expression and function of urate transporters, thereby influencing the processes of urate reabsorption and excretion.

Beyond their effects on purine-metabolizing enzymes, total saponins from *S. riparia* also modulate renal urate transport. At 500 mg/kg, total saponins from *S. riparia* significantly regulated renal urate transporters by decreasing URAT1 and increasing OAT1 protein expression, while exerting limited effects on GLUT9. Quantitative analysis showed that total saponins from *S. riparia* reduced URAT1 protein expression by approximately 12% and increased OAT1 expression by approximately 32% compared with the model group, whereas GLUT9 expression was only slightly reduced (approximately 5%). Co-administration with allopurinol further enhanced transporter regulation, resulting in approximately a 36% reduction in URAT1 expression, a 34% reduction in GLUT9 expression, and a 116% increase in OAT1 expression [74]. A similar direction of transporter regulation was observed with Pallidifloside D, a saponin glycoside constituent of *S. riparia.* When given alone at 5 mg/kg, pallidifloside D may also affect renal urate transport. Pallidifloside D reduced URAT1 and GLUT9 protein expression by approximately 7% and 6%, respectively, and increased OAT1 expression by approximately 14%. In combination with allopurinol, more pronounced alterations were observed, including reduced URAT1 and GLUT9 expression (approximately 23% and 33%, respectively) and increased OAT1 expression (approximately 82%) [76]. These findings indicate an enhanced response associated with combination treatment; however, whether this interaction represents true pharmacological synergism remains to be determined. Further studies have shown that smilaxchinoside A and smilaxchinoside C at 10 mg/kg significantly suppressed renal URAT1 protein expression. Quantitative analysis indicated that smilaxchinoside A and smilaxchinoside C [77] reduced URAT1 expression by approximately 13% and 12%, respectively, compared with the model group. Similarly, riparoside B and timosaponin J at the same dose decreased renal URAT1 expression by approximately 18% and 10%, respectively [77,78]. Collectively, these individual steroidal saponins act at distinct regulatory nodes involved in purine and uric acid metabolism, providing component-level evidence for the uric acid-lowering activity of total saponins from *S. riparia* and further elucidating the molecular basis of their multi-target action.

In addition to their effects on purine-metabolizing enzymes, gypenosides may also reduce SUA by modulating renal urate transport. After 6 weeks of administration, low-dose gypenosides (15 mg/kg) significantly lowered SUA levels in rats with HUA without significantly affecting the activities of the enzymes examined, suggesting that transporter-mediated mechanisms may also contribute to their urate-lowering action. At the 8-week endpoint, this low dose showed limited effects on purine-metabolizing enzymes but reduced renal GLUT9 expression by approximately 63%. By contrast, 60 mg/kg gypenosides exerted broader regulation of renal urate transporters, reducing URAT1 and GLUT9 expression by approximately 48% and 68%, respectively, while increasing OAT1 expression by approximately 124%. These transporter changes were accompanied by a 32.7% reduction in SUA, increased fractional excretion of uric acid, and amelioration of renal pathological injury at the 8-week endpoint [79]. These findings suggest that modulation of renal urate transport, ranging from a selective effect on GLUT9 at the lower dose to broader regulation of both reabsorptive and secretory transporters at the higher dose, may contribute to the urate-lowering effects of gypenosides.

*Dioscorea nipponica* Makino and *Dioscorea collettii* Hook. F. (Charuishuyu), both belonging to the Dioscoreaceae family, exhibit notable uric acid-lowering activity. Total saponins from *D. nipponica* and those from *D. collettii* each downregulate the expression of the urate reabsorption transporter URAT1 while upregulating the urate excretion transporters OAT1 and OAT3 [84,85], thereby reducing renal tubular urate reabsorption and facilitating urate excretion. For total saponins from *D. nipponica*, oral administration at 40 mg/kg for 7 days reduced SUA levels from 280.82 ± 51.85 to 220.24 ± 40.52 μmol/L, corresponding to an approximately 22% reduction. Western blot analysis showed a 35% decrease in renal URAT1 protein expression, accompanied by 44% and 33% increases in OAT1 and OAT3, respectively [84]. Total saponins from *D. collettii* additionally suppress URAT1 expression at both the mRNA and protein levels and reduce GLUT9 protein expression, thus inhibiting renal tubular urate reabsorption through multiple transporter targets. At 300 mg/kg, total saponins from *D. collettii* reduced renal URAT1 and GLUT9 protein expression by approximately 42% and 33%, respectively, while increasing OAT1 and OAT3 expression by approximately 31% and 25%. These coordinated changes in urate reabsorption and secretion transporters were accompanied by a reduction in SUA levels from 15.57 ± 4.85 to 12.15 ± 2.74 mg/dL after 28 days of treatment (approximately 22% decrease), potentially reducing renal tubular urate reabsorption and facilitating urate excretion through coordinated regulation of multiple transporter targets [85]. Moreover, Total saponins from *D. nipponica* also restored renal ABCG2 expression at both the mRNA and protein levels in PO-induced HUA mice. At the protein level, ABCG2 expression was increased in a dose-dependent manner, with the highest dose (600 mg/kg) producing an approximately 63% increase compared with the model group [86]. These findings suggest that upregulation of ABCG2 may represent an additional mechanism by which total saponins from *D. nipponica* facilitate renal urate excretion.

As a major bioactive component identified in Wuling Capsules, saikosaponin A may contribute to the regulation of urate transporters and the protection of renal tubular epithelial cells against uric acid-induced injury. In vitro studies showed that saikosaponin A (10 μM) significantly regulated the urate transporters in HK-2 cells exposed to 800 μg/mL. Quantitative analysis indicated that saikosaponin A reduced URAT1 and GLUT9 mRNA expression by approximately 70% and 57%, respectively, while increasing ABCG2 mRNA expression by approximately 322% [87]. These transporter-regulatory effects are accompanied by a significant improvement in cell viability and inhibition of apoptosis, with saikosaponin A producing a 28.71% inhibition of uric acid-induced HK-2 cell apoptosis, indicating that saikosaponin A not only promotes urate handling but also protects renal tubular epithelial cells against uric acid-induced injury.

The leaves of *Ilex cornuta* Lindl. & Paxton (Gougu) are traditionally used to dispel wind and dampness and are commonly prescribed for rheumatic pain, whereas the stems and leaves of *Achyranthes bidentata* Bl. (Niuxi) are used to dispel cold and dampness, strengthen the tendons and bones, and promote diuresis, particularly in conditions such as cold-damp paralysis, lower back and knee pain, and dysuria. Modern pharmacological studies have shown that saponins derived from both species downregulate the renal expression of the urate reabsorption transporters URAT1 and GLUT9 in animals with HUA, thereby suppressing renal urate reabsorption. Their effects on urate excretion transporters, however, differ. In *I. cornuta*, the crude saponin extract contained measurable amounts of total saponins, although individual saponin constituents and their relative abundance were not identified. Compared with other extracts, the saponin-enriched fraction exhibited preferential urate-lowering activity, supporting a potential contribution of saponin components to the observed effects. At doses of 50 and 100 mg/kg, the fraction reduced SUA by approximately 22% and 26%, respectively, compared with the model group. At 100 mg/kg, it decreased renal URAT1 and GLUT9 protein expression by approximately 53% and 67%, respectively, while increasing ABCG2 protein expression by approximately 162% [88]. Administration of total saponins from the stems and leaves of *A. bidentata* at 50, 100, and 200 mg/kg modulated renal urate transporters in HUA rats. Quantitative estimation from graphical data indicated that total saponins from the stems and leaves of *A. bidentata* reduced renal URAT1 and GLUT9 levels by approximately 10–25% and 5–20%, respectively, while increasing OAT1 expression by approximately 10–25%, with the most pronounced effects observed at 200 mg/kg. At 100 and 200 mg/kg, these transporter alterations were accompanied by significant reductions in SUA, which decreased by approximately 20% and 30%, respectively [89]. Through the coordinated inhibition of uric acid reabsorption and enhancement of urate excretion, these saponins reduce SUA levels, providing a modern pharmacological basis for the traditional therapeutic principles of “dispelling wind-dampness” and “promoting diuresis.”

A chemically characterized saponin-rich fraction from *Clematis chinensis* Osbeck (Weilingxian) exerts urate-lowering effects, at least in part, by modulating renal urate transporters. This active fraction contained two identified major oleanane-type triterpenoid saponins, including clematichinenoside AR2 at approximately 9.5% and a second triterpenoid saponin at approximately 2.7%. In hyperuricemic mice, administration of the fraction at 50 mg/kg significantly reduced SUA, with the high-dose treatment decreasing SUA by approximately 65%. Quantitative estimation indicated that the fraction reduced mRNA expression of the urate reabsorptive transporters URAT1 and GLUT9 by approximately 50%, while increasing the urate secretory transporters OAT3 and ABCG2 by approximately 104% and 43%, respectively. These findings suggest that coordinated regulation of renal urate reabsorption and secretion contributes to the urate-lowering activity of this fraction. The fraction also ameliorated renal injury, whereas allopurinol did not improve renal indices and was associated with aggravated renal damage in this model [90]. Subsequent work provided more direct compound-level evidence by isolating clematichinenoside AR, a structurally defined triterpenoid saponin with a purity exceeding 90%, from a related saponin fraction of *C. chinensis*. Clematichinenoside AR at 12.5–50 mg/kg exhibited a dose-dependent urate-lowering effect, reducing SUA by approximately 72–85%, with the greatest reduction observed at 50 mg/kg. Quantitative estimation indicated that high-dose treatment increased the expression of urate secretory transporters OAT1, OAT3, and ABCG2 mRNA by approximately 190%, 160%, and 169%, respectively. Increased OAT3 and ABCG2 protein expression was further confirmed by Western blotting [91]. These findings strengthen the evidence that chemically defined triterpenoid saponins contribute to the urate-lowering activity of *C. chinensis*.

Overall, saponins that regulate renal urate transporters appear to share a common pharmacological strategy of inhibiting urate reabsorption while promoting its excretion, although individual saponins differ in the spectrum of transporters they regulate and in their additional pharmacological effects. These characteristics provide a mechanistic basis for the development of saponin-based uric acid-lowering agents that target renal urate transport systems.

### 5.3. Regulation of the Gut Microbiota

The gut microbiota plays an important role in both purine metabolism and urate excretion. Increasing evidence indicates that saponin compounds can contribute to uric acid reduction by reshaping the intestinal microbial ecosystem. Their effects are mediated through several complementary mechanisms, including modulation of gut microbial composition, enhancement of SCFA production, restoration of intestinal barrier integrity, and regulation of intestinal urate transporter expression. Collectively, these actions improve intestinal uric acid handling and help restore gut microenvironmental homeostasis.

Saponins from *Astragalus membranaceus* (Fisch.) Bunge (Huangqi) appear to facilitate urate excretion and metabolism through bidirectional interactions with the gut microbiota. Owing to their relatively low oral bioavailability, a substantial proportion of these saponins remains within the intestinal lumen after administration, creating favorable conditions for direct interaction with resident microorganisms. The gut microbiota participates in the biotransformation of saponins from *A. membranaceus*. In vitro fermentation studies have shown that both *Lactobacillus acidophilus* and fecal microbiota can convert structurally related saponin precursors from *A. membranaceus* into astragaloside IV, whose concentration peaks after 12 h of co-culture before further metabolism into secondary products [92]. Further supporting this interaction, administration of an *A. membranaceus* preparation (1.5 g/kg, twice daily for 24 days) reduced SUA levels by approximately 30% in PO- and fructose-induced HUA rats. Metabolite profiling revealed substantial intestinal exposure to astragalus saponins and their transformation products, including astragalosides and cycloastragenol-derived metabolites. 16S rRNA sequencing showed that this treatment restored SCFAs-associated bacterial taxa, with the relative abundance of *Muribaculaceae* and *Clostridia* increased by approximately 100% and 130%, respectively, compared with the HUA model group, while *Lachnospiraceae* was restored toward normal levels. In addition, the abundance of the potentially pathogenic genus *Escherichia–Shigella* was reduced [93]. These findings suggest that intestinally exposed saponin constituents may contribute to the remodeling of gut microbiota associated with HUA improvement.

Ginseng is rich in dammarane-type tetracyclic triterpene saponins, among which ginsenoside Rg1 and several rare ginsenosides have demonstrated notable anti-hyperuricemic activity. In high-purine diet-induced HUA rats, oral administration of ginsenoside Rg1 (12.5 or 25 mg/kg/day for 8 weeks) significantly reduced SUA levels by approximately 49% and 55%, respectively. Beyond its urate-lowering effect, Rg1 reshaped gut microbial dysbiosis by increasing the abundance of beneficial bacteria, including *Ligilactobacillus*, *Lactobacillus*, *Ruminococcus*, and *Lachnospiraceae_NK4A136_group*. These microbial alterations were accompanied by enhanced SCFAs production (total SCFAs increased by approximately 29% and 45% after low- and high-dose Rg1 treatment, respectively) and reduced XOD activity. Beyond reshaping the gut microbial community, ginsenoside Rg1 enhances intestinal barrier integrity by upregulating the tight junction proteins ZO-1 and occludin, while ameliorating HUA-induced damage to intestinal villi and mucosal epithelial architecture. It also suppresses the expression of key inflammatory signaling proteins, including TRAF6 and NF-κB p65 [94], thereby attenuating intestinal inflammatory cascades and reducing secondary intestinal and renal injury associated with pro-inflammatory mediators.

Rare ginsenosides, mainly represented by Rg3S, Rg3R, F4, Rg6, Rk1, and Rg5 (>80% of total saponins), exert antihyperuricemic effects partly through gut microbiota remodeling. In PO-induced HUA mice, oral administration of rare ginsenosides (50, 100, or 200 mg/kg/day for 35 days) reduced SUA levels by approximately 25–42% in a dose-dependent manner. Concurrently, rare ginsenosides restored gut microbial diversity, corrected the elevated F/B ratio, and promoted the enrichment of beneficial bacteria, particularly Lactobacillus. Concurrently, rare ginsenosides restored microbial diversity and species richness, corrected the abnormally elevated F/B ratio, and promoted the enrichment of beneficial bacteria, particularly Lactobacillus. Multi-omics analysis further identified *Lactobacillus* and *Akkermansia* as core genera associated with rare ginsenosides intervention [95]. Through these coordinated changes in microbial composition and community structure, rare ginsenosides contribute to the restoration of gut microbiota homeostasis and the regulation of uric acid metabolism.

Total saponins from *D. nipponica* ameliorate gut microbial dysbiosis associated with HUA. In PO- and yeast diet-induced HUA rats, oral administration of total saponins from *D. nipponica* (40, 200, or 400 mg/kg for 14 days) significantly reduced SUA levels by approximately 34–62%, with the high-dose group decreasing SUA levels by approximately 40% compared with the model group. The urate-lowering effects of total saponins from *D. nipponica* were accompanied by marked remodeling of HUA-associated gut microbiota [96]. At the phylum level, they reduce the abnormally increased abundance of Bacteroidetes while increasing *Firmicutes* and *Campylobacterota*. At the genus level, they decrease potentially pathogenic taxa, including *Bacteroides* and *Parabacteroides*, while enriching beneficial bacteria such as *Lactobacillus*, *Clostridium*, and *Ruminococcus*. These changes restore the F/B ratio and enhance SCFAs production [96,97]. Notably, fecal microbiota transplantation further demonstrated that gut microbiota from donors treated with total saponins from *D. nipponica* reduced SUA levels in recipient mice [97], suggesting that the uric acid-lowering effect of these saponins is closely associated with gut microbiota modulation.

*Brassica rapa* L. (Qiamagu) is a medicinal and edible plant widely cultivated in Xinjiang, China. Total saponins from *B. rapa* have demonstrated anti-hyperuricemic activity in PO- and hypoxanthine-induced HUA mice. Oral administration at 500, 1000, or 1500 mg/kg/day for 14 days reduced SUA levels by approximately 15–50% across the tested doses. At the microbiota level, total saponins from *B. rapa* ameliorate HUA-associated gut microbiota dysbiosis by restoring the F/B ratio and reshaping the intestinal microbial community. Specifically, they enrich beneficial SCFAs-producing bacteria, including *Paramuribaculum* and *Duncaniella*, as well as uricase-producing taxa such as *Eubacterium_F* and *Companilactobacillus*, while reducing the abundance of the inflammation-associated genera *CAG-873* and *CAG-485*. These microbial changes are accompanied by alterations in fecal SCFA profiles, with acetate levels reduced by 16.9% and butyrate production increased by 40.5% compared with the HUA model group, thereby alleviating intestinal inflammation. In addition to remodeling the gut microbiota, total saponins from *B. rapa* upregulate intestinal ABCG2 expression [98], thereby promoting urate excretion and contributing to their uric acid-lowering effect.

A common mechanism by which saponins modulate the gut microbiota is the selective enrichment of beneficial bacteria accompanied by suppression of potentially harmful taxa. These microbial shifts contribute to restoration of intestinal barrier integrity and attenuation of inflammatory signaling, thereby establishing a coordinated cascade of microbiota rebalancing, barrier restoration, inflammation suppression, and enhanced uric acid clearance. Notably, individual saponins tend to favor the enrichment of distinct microbial taxa, suggesting that their microbiota-modulatory effects are not entirely uniform. This taxon-specific responsiveness may provide a basis for developing more precise gut microbiota-targeted intervention strategies for HUA and gout.

### 5.4. Regulation of Inflammatory Signaling Pathways

Saponins exert pronounced anti-inflammatory effects by modulating key signaling pathways involved in gout-associated inflammation. Through coordinated interference with inflammatory priming, activation, and downstream signal amplification, these compounds suppress the expression and release of pro-inflammatory mediators, thereby attenuating inflammatory injury in affected joints and surrounding tissues.

Total saponins from *D. nipponica* exert therapeutic effects against HUA and gout through coordinated modulation of multiple inflammatory signaling pathways. Within the NF-κB pathway, these total saponins downregulate TLR2 and TLR4, together with the downstream signaling molecules IRAK1, TRAF6, TAK1, and IKKα. They further inhibit IκBα phosphorylation and NF-κB p65 nuclear translocation, reduce NF-κB p65–DNA binding activity, and consequently suppress the transcription and release of pro-inflammatory cytokines, including IL-1β, IL-6, and TNF-α [99]. In parallel, in MSU-induced GA rats, total saponins from *D. nipponica* attenuated inflammatory responses by inhibiting NLRP3 inflammasome activation, as evidenced by downregulation of NLRP3, ASC, and cleaved caspase-1, thereby suppressing IL-1β maturation and release. Administration of total saponins from *D. nipponica* (160 mg/kg) reduced serum IL-1β levels from 39.43 ± 1.00 ng/L in the model group to 26.27 ± 0.87 ng/L, corresponding to an approximately 33% reduction. It also decreased inflammatory cytokine expression in synovial tissues, with TNF-α mRNA levels reduced from 1.32 ± 0.08 to 0.96 ± 0.06 (approximately 27% reduction). Furthermore, in MSU- and LPS-induced RAW264.7 macrophages, total saponins from *D. nipponica* reduced IL-1β levels in culture supernatants from 174.37 ± 5.30 ng/L to 50.04 ± 1.52 ng/L (approximately 71% reduction), further supporting inhibition of NLRP3 inflammasome-mediated inflammatory responses [100]. Their regulatory effects also extend to MAPK signaling, as reflected by decreased levels of p-MEK1/2, p-ERK1/2, and p-JNK, reduced MKK4 protein expression, and lower ICAM-1 and CXCL1 levels [101].

Further mechanistic studies have identified dioscin, a spirostanol-type steroidal saponin, as a key anti-inflammatory constituent of the total saponins from *D. nipponica*. Dioscin inhibits the TLR4/MyD88 signaling axis, decreases IKKβ phosphorylation, and suppresses both NF-κB p65 nuclear translocation and its DNA-binding activity. Simultaneously, it downregulates NLRP3, ASC, and caspase-1 protein expression and reduces the maturation and release of IL-1β. These regulatory effects were accompanied by reduced production of pro-inflammatory cytokines in MSU crystal-induced GA rats. At 50 mg/kg, dioscin decreased IL-1β, IL-6, and TNF-α levels by approximately 56%, 23%, and 67%, respectively; at 100 mg/kg, the corresponding reductions were approximately 65%, 66%, and 80% [102]. Dioscin also attenuates activation of the p38 MAPK pathway, inhibits MKK3/6 phosphorylation, and consequently reduces inflammatory cell infiltration and synovial hyperplasia [103]. Despite these anti-inflammatory effects, its potential hepatotoxicity warrants attention, as high-dose or prolonged administration may increase the risk of liver injury. Accordingly, the daily intake of total saponins should be carefully controlled in clinical use, with liver function monitored during long-term treatment [104].

Both ginsenoside Rb1 and ginsenoside Rg1 modulate gout-associated inflammatory signaling and thereby interrupt the amplification of inflammatory cascades, although their principal regulatory targets differ. In MSU crystal-induced GA rats, oral administration of ginsenoside Rb1 (80 mg/kg for 9 days) regulates inflammatory responses through modulation of NF-κB/NLRP3 signaling. Ginsenoside Rb1 suppresses IκBα degradation, inhibits NF-κB activation, and attenuates NLRP3 inflammasome activation. In joint tissues, these effects were accompanied by reduced transcription of pro-inflammatory cytokines, with IL-1β, IL-6, and TNF-α mRNA levels decreased by approximately 47%, 47%, and 41%, respectively [105]. In contrast to the NF-κB/NLRP3-centered regulation of ginsenoside Rb1, ginsenoside Rg1 exerts broader modulation of upstream inflammatory signaling pathways. In MSU crystal-induced acute gouty arthritis models, administration of ginsenoside Rg1 (12.5 or 25 mg/kg for 7 days) modulates TLR4/MyD88/NLRP3 and MAPK signaling pathways. Ginsenoside Rg1 downregulates TLR4, MyD88, NLRP3, ASC, and caspase-1 expression, thereby suppressing TLR4/MyD88-dependent inflammatory priming and interfering with NLRP3 inflammasome activation. These regulatory effects were accompanied by reduced pro-inflammatory cytokine levels in synovial tissues, with IL-1β decreased by approximately 21% and 17% and IL-6 decreased by approximately 34% and 27% at 12.5 and 25 mg/kg, respectively. In addition, ginsenoside Rg1 inhibits the phosphorylation of p38 MAPK, ERK, and JNK, thereby weakening MAPK-mediated promotion of NLRP3 inflammasome activation. This, in turn, suppresses NET formation and attenuates the inflammatory response in acute gouty arthritis [106].

In MSU crystal-induced GA rats, pretreatment with madecassoside (10, 20, or 40 mg/kg/d for two weeks before MSU challenge) attenuated inflammatory responses through regulation of NF-κB/NLRP3 signaling pathways. Madecassoside suppresses activation of the NF-κB signaling pathway by downregulating NF-κB at both the mRNA and protein levels. Concurrently, it reduces the expression of NLRP3 inflammasome-associated components, including NLRP3, ASC, and caspase-1 [107], as well as neutrophil cytoplasmic factor-1, thereby inhibiting NLRP3 inflammasome assembly and activation and limiting neutrophil recruitment [108]. Consistent with these molecular alterations, high-dose madecassoside reduced serum IL-1β levels from 3.19 ± 1.11 ng/L in the model group to 2.39 ± 1.05 ng/L (approximately 25% reduction) and decreased TNF-α levels from 9.55 ± 1.50 ng/L to 8.77 ± 1.29 ng/L (approximately 8% reduction) [107]. These anti-inflammatory effects ultimately resulted in attenuation of MSU crystal-induced joint swelling and histopathological injury.

Anemoside B4 exerts anti-inflammatory effects by simultaneously suppressing NF-κB signaling and inhibiting NLRP3 inflammasome assembly and activation. At the priming stage, it reduces the phosphorylation of IKKα/β, IκBα, and NF-κB p65, prevents NF-κB p65 nuclear translocation, and consequently downregulates the transcription of NLRP3 and pro-IL-1β. At the activation stage, Anemoside B4 suppresses MSU crystal-induced ROS production in macrophages, alleviates mitochondrial damage, and thereby interrupts upstream signals required for inflammasome activation. In addition, it specifically binds to NIMA-related kinase 7 (NEK7), a key kinase involved in NLRP3 activation, disrupting the NEK7–NLRP3 interaction and directly interfering with inflammasome assembly. In MSU crystal-induced GA mice, administration of Anemoside B4 (5, 10, or 20 mg/kg/d for 7 days) reduced pro-inflammatory cytokine levels in ankle edema tissue homogenates, decreasing IL-1β, IL-6, and TNF-α levels by approximately 12–14%, 13–30%, and 6–8%, respectively, compared with the MSU model group [109]. Through these coordinated actions, Anemoside B4 attenuated gout-associated inflammatory responses.

Bixie is the dried rhizome of *Dioscorea hypoglauca* Palibin, a member of the Dioscoreaceae family, and is traditionally used to drain dampness and turbidity and dispel wind-dampness. In MSU-induced inflammatory models, total saponins from *D. hypoglauca* exhibited anti-inflammatory activity at both cellular and animal levels. In MSU-stimulated THP-1 macrophages, treatment with total saponins from D. hypoglauca (1, 3, or 10 mg/L) suppressed activation of the TLR4/MyD88/NF-κB signaling pathway by downregulating TLR4 and NF-κB expression at both the mRNA and protein levels and inhibiting NF-κB p65 nuclear translocation [110]. In parallel, in MSU crystal-induced acute gouty arthritis rats, administration of total saponins from D. hypoglauca (30, 100, or 300 mg/kg for 7 days) inhibited NLRP3 inflammasome activation by reducing the expression of NLRP3, ASC, and pro-caspase-1 and suppressing pro-caspase-1 cleavage and activation. These effects were accompanied by decreased serum inflammatory cytokine levels, with IL-1β reduced by approximately 12%, 24%, and 35% and TNF-α reduced by approximately 7%, 24%, and 45% at 30, 100, and 300 mg/kg, respectively [111]. These findings indicate that total saponins from *D. hypoglauca* attenuate gout-associated inflammation through inhibition of NLRP3 inflammasome activation.

Total saponins from *Panax notoginseng* (Burk.) F.H. Chen (Sanqi) and *Platycodon grandiflorus* (Jacq.) A. DC. (Jiegeng), together with the individual saponins clematichinenoside AR and Doliroside A, exert anti-gout effects by targeting the NLRP3 inflammasome signaling pathway [91,112,113,114]. These saponin components inhibited NLRP3 inflammasome activation by downregulating key inflammasome-related proteins, including NLRP3, ASC, pro-caspase-1, and caspase-1, thereby interfering with inflammasome assembly and caspase-1 activation. These molecular changes were accompanied by differential regulation of inflammatory cytokines. Total saponins from *P. notoginseng* reduced serum IL-1β and TNF-α levels, with high-dose treatment decreasing IL-1β and TNF-α by approximately 29% and 13%, respectively [112]. Total saponins from *P. grandiflorus* preferentially suppressed TNF-α production, reducing TNF-α by approximately 18–27%, whereas IL-1β and IL-6 showed relatively modest reductions [113]. Compared with the MSU crystal-treated group, Doliroside A (10, 20, and 40 mg/kg) reduced the MSU crystal-induced elevation of serum IL-1β by approximately 21–55% and that of serum TNF-α by approximately 18–41% [114], while clematichinenoside AR decreased IL-1β, IL-6, and TNF-α levels by approximately 45–53%, 14–46%, and 58–63%, respectively [91]. Additional mechanisms further broaden their anti-inflammatory effects. Time-course administration experiments with Doliroside A suggest that it interferes with NF-κB-mediated transcriptional priming of pro-IL-1β and reduces prostaglandin E_2_ (PGE_2_) levels, thereby alleviating joint pain [114]. Total saponins from *P. notoginseng* also suppress MSU-induced ICAM-1 expression and vascular endothelial cell apoptosis [115], whereas clematichinenoside AR alleviates oxidative stress through activation of the NRF2/HO-1 signaling pathway [91]. These findings indicate that the anti-inflammatory actions of saponins derived from CMMs extend beyond inflammasome inhibition to encompass antioxidant and vascular protective effects.

*Smilax nipponica* Miq. (Baibei Niuweicai), a closely related species of Niuweicai within the same genus, likewise contains saponin constituents with anti-gout activity. In MSU crystal-induced GA rats, administration of total saponins from *S. nipponica* (85 or 340 mg/kg) reduced serum levels of pro-inflammatory cytokines. Compared with the model group, IL-1β levels decreased by approximately 30% and 36%, IL-6 levels decreased by approximately 28% and 35%, and TNF-α levels decreased by approximately 9% and 19% at the two doses, respectively. Further phytochemical investigation led to the isolation of two steroidal saponins, smilnipponicoside A and smilnipponicoside C. In MSU-stimulated RAW264.7 cells, both compounds directly reduce the levels of PI3K, p-PI3K, and p-Akt and decrease the p-PI3K/PI3K and p-Akt/Akt ratios [116], thereby suppressing PI3K/Akt signaling and interrupting downstream inflammatory signal transduction. These findings identify smilnipponicosides A and C as important bioactive constituents underlying the anti-gout effects of total saponins from *S. nipponica*.

The anti-inflammatory effects of saponins derived from CMMs are not confined to the inhibition of a single signaling pathway; rather, they arise from coordinated regulation of multiple molecular targets and interconnected inflammatory pathways. Current mechanistic studies have focused predominantly on the NLRP3 inflammasome pathway. Notably, certain saponins concurrently modulate key regulatory nodes across several signaling cascades, thereby interrupting inflammatory priming, activation, and downstream signal amplification at multiple levels. Such multi-target and multi-pathway actions may enable more comprehensive suppression of the gout-associated inflammatory cascade than interventions directed against a single signaling pathway.

## 6. Comparative Analysis of the Pharmacological Effects of CMM-Derived Saponins in HUA and Gout

The preceding sections have summarized the therapeutic mechanisms of CMM-derived saponins against HUA and gout, including regulation of purine metabolism, renal urate transport, gut microbiota, and inflammatory signaling. However, although numerous studies have demonstrated significant pharmacological activities, direct quantitative comparison of therapeutic efficacy among different saponins remains challenging because of considerable heterogeneity in experimental conditions, including animal species, disease induction methods, administered doses, treatment durations, and evaluated endpoints. Therefore, the available evidence should be interpreted as comparative pharmacological responses and therapeutic characteristics rather than definitive efficacy rankings. In addition, because complete numerical values were not consistently reported in all original studies, some efficacy parameters summarized in Table 2 were estimated from graphical presentations using WebPlotDigitizer v3.4 and should therefore be considered approximate indicators of response magnitude rather than precise quantitative measurements.

### 6.1. Comparative Pharmacological Profiles of Saponins Targeting Purine Metabolism-Related Enzymes

Among the steroidal saponins isolated from *S. riparia*, four representative compounds showed different hypouricemic activities under comparable experimental conditions. Riparoside B exhibited the strongest activity among these compounds, showing relatively greater inhibition of XOD activity and reduction in SUA levels (approximately 23%) [78]. However, the relationship between XOD inhibition and SUA reduction was not completely consistent among different saponins. Although Flaccidoside II showed weaker XOD inhibition than riparoside B, it achieved a greater reduction in SUA levels (approximately 26.1%) at a relatively low dose of 8 mg/kg [83], suggesting that additional factors beyond XOD inhibition may contribute to its hypouricemic effect. Compared with individual steroidal saponins, some total saponin preparations showed considerable hypouricemic effects under their respective experimental conditions. Total saponins from *D. nipponica* significantly reduced SUA levels in hyperuricemic mice in a dose-dependent manner, with reductions of approximately 29.4% and 43% at 60 and 600 mg/kg, respectively [81]. Notably, high-dose treatment further enhanced the hypouricemic effect and was accompanied by decreased serum XOD and ADA activities. Similarly, gypenosides reduced SUA levels by approximately 32.7% after long-term administration at 60 mg/kg, accompanied by regulation of multiple purine metabolism-related enzymes [79]. These findings suggest that certain total saponin preparations may exert hypouricemic effects through broader regulation of purine metabolism-related enzymes. Although these total saponin preparations exhibited favorable hypouricemic activity, their efficacy should be interpreted together with their higher administered doses and longer treatment duration compared with individual saponins.

### 6.2. Comparative Efficacy of Saponins Targeting Urate Transporters

Compared with enzyme-targeting studies, direct efficacy comparisons among transporter-regulating saponins remain limited due to differences in experimental models, doses, and treatment durations. Therefore, their pharmacological effects are better evaluated based on transporter regulation patterns and associated hypouricemic responses.

Four steroidal saponins derived from *S. riparia* mainly modulated renal urate reabsorption by suppressing renal URAT1 expression. Under comparable conditions, these compounds showed moderate SUA-lowering effects; riparoside B exhibited relatively stronger activity, which was accompanied by an approximately 18% reduction in URAT1 expression [78]. Overall, individual steroidal saponins mainly exert urate-lowering effects through inhibition of renal urate reabsorption, whereas some total and triterpenoid saponin preparations regulate both urate reabsorption and excretion pathways.

In contrast, some total saponin preparations exhibited broader regulation of urate transport networks. Total saponins from *D. nipponica* and gypenosides modulated multiple transporters, including reductions in URAT1/GLUT9 and increases in OAT1, OAT3, and ABCG2 protein expression, which were associated with decreased SUA levels [79,84]. Certain triterpenoid saponins, such as clematichinenoside AR, further enhanced urate excretion by regulating OAT1, OAT3, and ABCG2 [91]. These findings suggest that multi-transporter regulation may represent an important feature of some saponin preparations.

Overall, individual steroidal saponins mainly exert urate-lowering effects through modulation of renal urate reabsorption, whereas some total and triterpenoid saponin preparations influence both urate reabsorption and excretion pathways. However, standardized comparative studies are still required to determine the relative pharmacological potency of different saponins.

### 6.3. Comparative Pharmacological Profiles of Saponins Targeting Inflammatory Signaling Pathways

The anti-inflammatory effects of saponins derived from CMMs are not confined to the inhibition of a single signaling pathway; rather, they arise from coordinated regulation of multiple molecular targets and interconnected inflammatory pathways.

Among the individual saponins evaluated, dioscin and clematichinenoside AR showed pronounced cytokine-suppressive profiles. Dioscin reduced IL-1β, IL-6, and TNF-α levels by approximately 56–65%, 23–66%, and 67–80%, respectively [102], whereas clematichinenoside AR decreased these cytokines by approximately 45–53%, 14–46%, and 58–63%, respectively [91]. Doliroside A also demonstrated notable anti-inflammatory activity [114], while madecassoside and anemoside B4 showed relatively moderate cytokine suppression under the tested conditions [109]. Within the ginsenoside family, Rb1 produced greater cytokine suppression under its tested conditions, reducing IL-1β, IL-6, and TNF-α expression by approximately 47%, 47%, and 41%, respectively [105], whereas Rg1 exhibited broader regulation of upstream inflammatory pathways [106]. Among total saponin preparations, those from *D. nipponica*, *D. hypoglauca*, and *S. nipponica* demonstrated consistent anti-inflammatory potential through regulation of inflammatory signaling pathways [100,111,116]. Direct comparability across these studies is constrained by substantial dose variation (5–300 mg/kg), differing treatment durations, and the use of distinct biological matrices for cytokine measurement. Overall, CMM-derived saponins exhibit diverse anti-inflammatory profiles, with differences in cytokine suppression and pathway regulation depending on their chemical structures and regulatory targets.

The comparative analysis indicates that CMM-derived saponins exhibit diverse pharmacological profiles depending on their chemical structures and regulatory targets. Individual saponins often display relatively selective activities toward specific pathways, such as XOD inhibition, URAT1 modulation, or inflammatory signaling suppression, whereas some total saponin preparations may exert broader effects through simultaneous regulation of multiple metabolic and inflammatory processes. However, differences in experimental models, doses, and treatment durations currently prevent reliable efficacy ranking among different saponins. Moreover, the comparability of pharmacological effects varies among different mechanistic categories. This limitation is particularly evident in gut microbiota-related studies, where heterogeneity in experimental designs and microbiota profiling approaches currently hampers direct pharmacological comparisons. Current evidence supports the contribution of gut microbiota remodeling to the hypouricemic actions of saponins, while its role in determining efficacy differences among individual compounds remains to be clarified. Future studies using standardized experimental designs and head-to-head comparisons are required to clarify the structure–activity relationships and pharmacological characteristics of specific saponin components.

## 7. Chemical Structures and Structure–Activity Relationships Underlying the Hypouricemic and Anti-Gout Effects of Saponins

**Figure 5:**
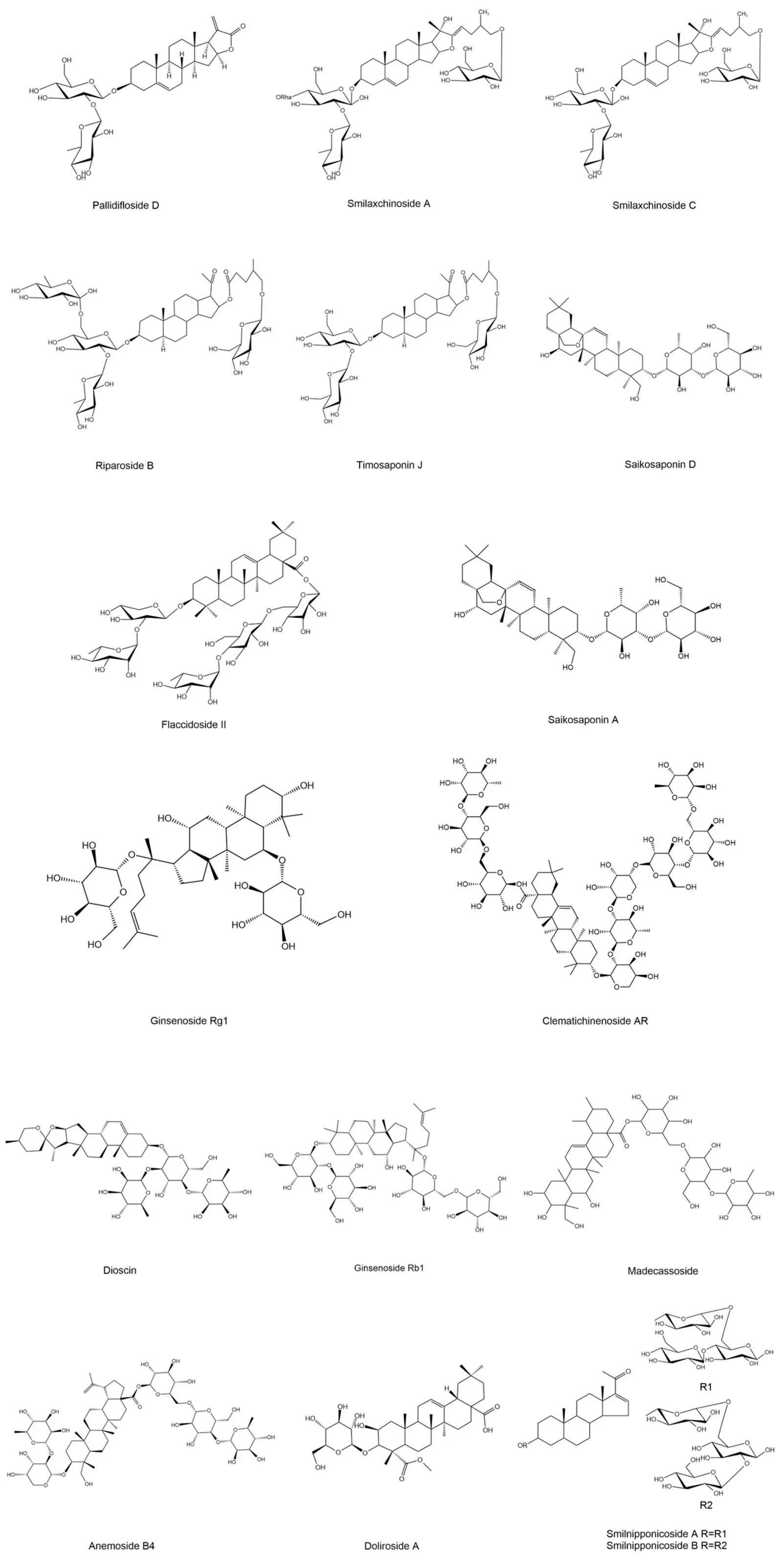
Chemical structures of the related saponin monomers.

The pharmacological activities of saponins described in the preceding sections are ultimately determined by their chemical structures, including aglycone skeleton type, glycosylation pattern, and sugar chain composition. To facilitate the structure–activity relationship analysis presented in the following subsections, the chemical structures and key structural features of the saponin compounds discussed in this review are summarized in Figure 5 and Table 3.

### 7.1. Structure–Activity Relationship of Saponins in the Regulation of Purine-Metabolizing Enzymes

Available evidence suggests that both aglycone structure and glycosylation may influence the regulation of purine-metabolizing enzymes, with XOD being the most extensively studied target. Smilaxchinoside A, Smilaxchinoside C, Riparoside B, and Timosaponin J differ in their steroidal scaffolds and sugar moieties, but all reduce serum or hepatic XOD activity [77,78], indicating that this effect is not confined to a single glycosylation pattern. Notably, Smilaxchinoside A, which contains an additional α-L-rhamnopyranosyl-(1 → 4) residue at C-3 relative to Smilaxchinoside C, shows slightly stronger inhibition of serum XOD activity, suggesting that C-3 sugar-chain variation may modulate activity [77]. Glycosylation also appears relevant to Flaccidoside II. Flaccidoside II primarily suppresses hepatic XOD activity at relatively high doses, thereby reducing uric acid, whereas its aglycone oleanolic acid and partially deglycosylated derivatives exhibit markedly attenuated hypouricemic activity [83]. Meanwhile, Saikosaponin D and Pallidifloside D exhibit different enzyme-regulatory profiles involving ADA/XOD and XOD/PRPS/HGPRT/PRPPAT, respectively [75,80]. Thus, aglycone and glycosylation characteristics may jointly shape enzyme-regulatory activity, although a definitive structure–activity relationship (SAR) remains to be established.

### 7.2. Structure–Activity Relationship of Saponins in the Regulation of Urate Transporters

Structural differences among saponins are also associated with distinct urate-transporter profiles. Several Smilax-derived steroidal saponins, including Smilaxchinosides A and C, Riparoside B, and Timosaponin J, consistently downregulate URAT1 despite differences in scaffold and glycosylation [77,78], whereas Pallidifloside D additionally affects GLUT9 and OAT1 [76]. The slightly stronger URAT1 regulation observed for Smilaxchinoside A than for Smilaxchinoside C further suggests a possible contribution of C-3 sugar-chain composition. Triterpenoid saponins appear to involve a broader range of transporters: Saikosaponin A regulates URAT1, GLUT9, and ABCG2 [87], whereas Clematichinenoside AR predominantly enhances OAT1, OAT3, and ABCG2 expression [91]. These observations support a preliminary association between saponin structure and transporter-regulatory profile, although the structural determinants of transporter preference remain unresolved.

### 7.3. Structure–Activity Relationship of Ginsenosides in the Regulation of Gut Microbiota

The microbiota-regulatory effects of ginsenosides may be influenced by their glycosylation status and structural transformation. Ginsenoside Rg1, a PPT-type saponin glycosylated at C-6 and C-20, alleviates HUA-associated gut dysbiosis and increases *Lactobacillus*, *Ruminococcus*, and *Lachnospiraceae_NK4A136_group*, together with enhanced SCFA production. In contrast, rare ginsenosides, including Rg3(S), Rg3(R), F4, Rg6, Rk1, and Rg5, are characterized by reduced glycosylation and further structural transformation and mainly affect *Lactobacillus* and *Akkermansia*. Notably, *Lactobacillus* was commonly modulated by both Rg1 and the rare ginsenosides. These findings suggest a possible association between ginsenoside structural variation and microbiota responses, although direct SAR evidence from structurally matched monomers remains limited.

### 7.4. Structure–Activity Relationship of Saponins in the Regulation of Inflammatory Signaling Pathways

Structurally diverse steroidal and triterpenoid saponins exhibit partially overlapping anti-inflammatory effects, with NF-κB/NLRP3-related signaling representing a recurrent pharmacological target. However, differences in aglycone subclass and glycosylation pattern may be associated with distinct signaling profiles. This is illustrated by ginsenosides Rb1 and Rg1, which share a dammarane scaffold but differ in aglycone subclass and glycosylation. Rb1 is a PPD-type ginsenoside bearing sugar chains at C-3 and C-20 and primarily modulates NF-κB/NLRP3 signaling [105], whereas Rg1, a PPT-type ginsenoside glycosylated at C-6 and C-20, additionally affects TLR4/MyD88 and MAPK signaling [106]. These differences suggest a possible association between structural variation within the dammarane class and inflammatory signaling profiles, although direct head-to-head comparisons are lacking.

Differences in target profiles are also observed across more structurally divergent saponins. Anemoside B4, a lupane-type pentacyclic triterpenoid saponin, directly interferes with NEK7–NLRP3 inflammasome assembly [109], whereas the pregnane-type steroidal glycosides Smilnipponicosides A and C are primarily associated with PI3K/Akt regulation [116]. Meanwhile, structurally distinct saponins such as madecassoside and Doliroside A also converge on NF-κB/NLRP3-related inflammatory responses [107,114]. Thus, aglycone and glycosylation characteristics may contribute to differences in pathway and target profiles, but current evidence is insufficient to assign individual structural features to specific inflammatory targets.

### 7.5. Overall Structural Determinants Underlying the Biological Activities of Saponins

Taken together, the available data indicate that the hypouricemic and anti-gout activities of saponins are unlikely to depend on a single structural element. The aglycone scaffold appears to define the basic structural context for biological activity, whereas the number, position, and arrangement of sugar moieties may further modify activity profiles. Preliminary trends can be recognized across purine-metabolizing enzymes, urate transporters, gut microbiota, and inflammatory signaling; however, most evidence derives from individual compounds or mixtures rather than matched structural analogues. Accordingly, current findings are better regarded as structure–activity associations rather than definitive structure–activity rules, and systematic comparisons of compounds differing in defined aglycone or glycosylation features will be required to establish more robust structure–activity relationships.

## 8. Pharmacokinetics and Bioavailability of Saponins

### 8.1. General Pharmacokinetic Characteristics and Factors Limiting Oral Bioavailability

Despite the broad pharmacological activities described above, the extent to which saponins can reach their sites of action after oral administration is strongly influenced by their generally limited and variable bioavailability. This pharmacokinetic limitation arises from the combined effects of unfavorable physicochemical properties and multiple biological barriers. The relatively large molecular size and abundant glycosyl residues of many saponins increase their hydrogen-bonding capacity, polar surface area, and molecular flexibility, thereby limiting intestinal membrane permeability despite potentially favorable aqueous solubility [123,124]. Intestinal absorption may be further restricted by efflux transporters in certain saponins, whereas their relatively slow absorption and prolonged gastrointestinal residence increase the likelihood of microbial hydrolysis and deglycosylation before systemic absorption [123]. Following absorption, rapid hepatobiliary elimination may further reduce systemic exposure [124]. Collectively, poor membrane permeability, presystemic biotransformation, transporter-mediated efflux, and biliary elimination contribute to the low and variable oral bioavailability observed for many saponins.

### 8.2. Pharmacokinetic Characteristics and Bioavailability of Representative Saponins Relevant to HUA and Gout

Available pharmacokinetic studies indicate considerable variability in the systemic exposure of individual saponins after oral administration. To ensure comparability, only studies in which individual saponins were directly administered were included, whereas those using herbal extracts, total saponin fractions, compound preparations, or modified formulations were excluded from the primary comparison. However, pharmacokinetic characterization remains uneven among the compounds discussed in this review, with relatively detailed data available for only a limited number of representative saponins. The available pharmacokinetic parameters are summarized in Table 4.

Among the saponins for which absolute oral bioavailability has been determined, saikosaponin A showed particularly limited systemic availability, with an F value of approximately 0.04% across oral doses of 50–200 mg/kg [126]. Similarly, the absolute oral bioavailability of dioscin was only 0.21–0.23% [130], whereas that of ginsenoside Rb1 was 0.78% [129], and anemoside B4 exhibited an oral bioavailability of <1% [132]. Systemic exposure nevertheless varied considerably among compounds, as reflected by marked differences in C_max_ and AUC. Dioscin was also characterized by a delayed Tmax and a relatively long elimination half-life, suggesting prolonged absorption and slower systemic elimination. Because the administered doses and experimental designs differed substantially among studies, these pharmacokinetic parameters should be interpreted as compound-specific exposure profiles rather than used for direct cross-compound potency comparisons.

Biotransformation may further alter pharmacologically relevant exposure by generating metabolites with biological activities distinct from those of the parent compounds. Dioscin provides a representative example because of its extremely low oral bioavailability. Following oral administration, diosgenin, tigogenin, and sarsasapogenin have been identified as its major metabolites. Among these metabolites, tigogenin inhibited URAT1-mediated urate uptake, whereas both tigogenin and diosgenin enhanced ABCG2-dependent transcellular urate transport, suggesting that biotransformation of dioscin may contribute to the regulation of urate handling after oral administration [133]. Collectively, these observations indicate that the pharmacological effects of orally administered dioscin may involve contributions from both the parent compound and its bioactive metabolites, rather than being solely explained by systemic exposure to unchanged dioscin. However, direct evidence linking saponin-derived metabolites to anti-hyperuricemic or anti-gout effects remains limited, and the pharmacodynamic contribution of active metabolites has yet to be established for most saponins discussed in this review.

Importantly, direct quantitative comparison between in vivo exposure and the concentrations required for the mechanistic effects described above remains difficult for most saponins because tissue concentrations, unbound exposure, and metabolite exposure have rarely been characterized in parallel with pharmacodynamic responses. Therefore, whether all reported molecular effects can be achieved through direct systemic exposure to the parent saponins remains uncertain, particularly for compounds with very low oral bioavailability.

## 9. Safety and Toxicity of Saponins

Although saponins have shown promising therapeutic effects in hyperuricemia and gout through urate-lowering, gut microbiota-regulating, and anti-inflammatory activities, their safety profiles require further evaluation. Current evidence suggests that saponin toxicity is compound-specific and influenced by structural characteristics, dosage, exposure duration, and administration conditions. Therefore, safety assessment should be interpreted according to individual saponin components and experimental contexts.

Among total saponin preparations, hepatic injury has emerged as a recurrent concern under high-dose exposure. Total saponins from *D*. *nipponica* were generally well tolerated in acute and short-term studies, but repeated high-dose administration produced reversible increases in alanine aminotransferase (ALT) and mild hepatic hydropic changes, indicating a clear dose-related hepatic response [104]. A similar dose-related hepatic liability was observed with total saponins from *P*. *notoginseng* during prolonged oral exposure. In rats, 13-week gavage administration at 1.6 g/kg increased serum aspartate aminotransferase (AST) and ALT levels and relative liver weight, accompanied by hepatocellular vacuolar degeneration; the abnormal biochemical indices returned to normal after a 2-week recovery period [134].

The toxicological characteristics of individual saponins are better defined. Saikosaponin A and saikosaponin D are representative hepatotoxic compounds. Repeated oral exposure to saikosaponin A in mice produced dose-dependent hepatic changes together with activation of endoplasmic reticulum stress and autophagy-related pathways [135]. Saikosaponin D likewise showed concentration-dependent toxicity in LO2 hepatocytes, with an IC_50_ of approximately 2.14 μM, and has also been associated with cardiotoxic, neurotoxic, and hemolytic effects in other experimental settings [136]. Dioscin has been more extensively examined under repeated exposure. In vitro, it caused hepatocyte injury and apoptosis, whereas prolonged high-dose administration in rats was associated mainly with reduced food intake, gastrointestinal dysfunction, and mild hematological alterations [137]. These findings indicate that both exposure level and duration are important determinants of saponin toxicity.

By contrast, several monomeric saponins have shown comparatively favorable short-term tolerability. Flaccidoside II produced no mortality or overt toxic signs after a single high oral dose in mice, although repeated-dose and chronic toxicity data remain lacking [83]. Anemoside B4 also showed limited short-term toxicity [138], with no apparent cytotoxicity in HEK293 cells and no significant changes in body weight, blood urea nitrogen, or serum creatinine after high-dose administration in mice. Madecassoside displayed low cytotoxicity in HaCaT cells and low acute toxicity in zebrafish within the tested ranges, although adverse effects emerged at higher concentrations [139,140]. These findings support relatively favorable acute or short-term tolerability but do not establish long-term safety.

Developmental toxicity also deserves attention for certain ginsenosides. Ginsenoside Rg1 impaired embryonic growth and morphological development in rat and mouse whole-embryo culture models [141]. Ginsenoside Rb1 similarly affected early embryonic development [142]; exposure of mouse blastocysts increased apoptosis and reduced inner cell mass and trophectoderm cell numbers, with subsequent impairment of developmental potential. These observations are derived from embryonic models and therefore should be interpreted as developmental safety signals rather than evidence of general systemic toxicity. The toxicological findings of representative saponins and total saponin extracts are summarized in Table 5.

Taken together, hepatotoxicity is the most consistently documented adverse effect among the saponins examined, particularly for saikosaponins, dioscin, and some high-dose total saponin preparations. Evidence for nephrotoxicity is more limited and remains largely confined to specific high-dose experimental settings. More importantly, favorable acute or short-term tolerability does not substitute for long-term safety assessment. For compounds such as Flaccidoside II, Anemoside B4, and Madecassoside, repeated-dose and chronic toxicity data remain insufficient. Future studies should therefore prioritize clinically relevant exposure regimens and systematic evaluation of hepatic, renal, and developmental safety to define reliable safety margins for saponin-based interventions.

## 10. Conclusions and Future Perspectives

This review summarizes the major sources of saponin-type compounds derived from Chinese medicinal materials reported in recent years and systematically examines their pharmacological mechanisms in the treatment of HUA and gout. Overall, these mechanisms converge on two principal therapeutic dimensions: urate lowering and inflammation regulation. With respect to urate lowering, saponins suppress uric acid production by inhibiting key purine-metabolizing enzymes, including XOD and ADA; modulate the expression of urate transporters to reduce renal uric acid reabsorption and promote its excretion; and reshape the gut microbial community while enhancing the production of SCFAs, thereby facilitating intestinal uric acid clearance. Together, these coordinated actions establish a multidimensional urate-lowering network characterized by reduced uric acid production, enhanced degradation and excretion, and intestinal–renal cooperation.

In parallel, saponins exert anti-inflammatory effects through multiple complementary mechanisms. Restoration of intestinal barrier integrity helps alleviate persistent inflammatory stimulation associated with gut-derived factors, whereas direct modulation of key signaling pathways, including NF-κB, the NLRP3 inflammasome, and MAPK signaling, interferes with inflammatory priming, inflammasome assembly and activation, and downstream cascade amplification, ultimately reducing the production and release of pro-inflammatory mediators. Certain saponins further regulate the balance between M1 and M2 macrophage polarization, inhibit the formation of NETs, and attenuate MSU crystal-induced pain. Collectively, these findings indicate that saponins derived from Chinese medicinal materials exert therapeutic effects against HUA and gout through coordinated regulation of uric acid homeostasis and inflammatory responses, reflecting the characteristic multi-target and multi-pathway pharmacological profile of this class of compounds.

Current evidence indicates considerable heterogeneity in both the breadth and depth of pharmacological action among different saponin preparations and individual saponins. The mechanisms of saponins derived from several Chinese medicinal materials, including *Smilax riparia* A. DC., *Dioscorea nipponica* Makino, ginseng, *Gynostemma pentaphyllum* (Thunb.) Makino, have been investigated relatively systematically. Their pharmacological effects extend across multiple pathological processes, encompassing regulation of purine-metabolizing enzymes and urate transporters, remodeling of the gut microbiota, and coordinated modulation of inflammatory signaling pathways. This broad spectrum of activity highlights the multi-target therapeutic characteristics of saponins and supports their further mechanistic investigation and development. However, the therapeutic potential of saponins is still supported mainly by preclinical evidence. Among the 41 mechanism-related studies included in this review, 37 were based on in vivo animal models, 1 used an in vitro model, and 3 combined both approaches. Given that clinical validation in humans remains limited, further well-designed clinical trials are needed to determine whether these preclinical findings can be translated into meaningful therapeutic benefits.

Among these preparations, total saponins from *S. riparia* exhibit notable uric acid-lowering activity. Several active individual saponins have been isolated and identified, providing a clearer component basis for their pharmacological effects. These compounds act on key enzymes and transporters involved in uric acid metabolism and thereby cooperatively reduce uric acid levels; moreover, enhanced urate-lowering effects have been observed when they are combined with allopurinol, although formal synergy analysis remains to be performed. Their relatively well-defined pharmacological basis therefore provides substantial potential for the development of novel uric acid-lowering agents.

Total saponins from *D. nipponica*, in contrast, display a broader spectrum of activity encompassing uric acid reduction, gut microbiota modulation, and regulation of inflammatory signaling pathways. Dioscin, identified as a core active component, has been confirmed to constitute an important pharmacological basis for their anti-inflammatory effects. Collectively, these independent studies indicate that total saponins from *D. nipponica* may affect urate metabolism, gut microbiota, and inflammatory signaling through multiple mechanisms. However, these effects were demonstrated in separate experimental settings, and their simultaneous contribution has not yet been established. Among the saponin preparations investigated to date, total saponins from *D. nipponica* Makino have been studied across a particularly broad range of mechanisms and in considerable depth, suggesting substantial translational potential and promising prospects for new drug development. Ginsenoside Rg1 is likewise notable for its dual regulatory effects on intestinal barrier restoration and inflammatory signaling. By comparison, mechanistic studies of Flaccidoside II, Doliroside A, Madecassoside, and several other saponins remain largely confined to individual targets or signaling pathways. Whether these compounds can coordinately regulate the broader pathological network linking uric acid metabolism, gut microbiota dysbiosis, and inflammatory signaling has yet to be fully elucidated.

Despite these advances, several limitations continue to constrain research in this field. First, most studies have focused on total saponin fractions, whereas the specific pharmacologically active constituents, dose–response relationships, and structure–activity relationships of individual saponins remain insufficiently characterized. Second, many saponins exhibit relatively low oral bioavailability, and their complex absorption, distribution, and metabolic processes in vivo may limit the full expression of their pharmacological effects. Third, the current evidence base is dominated by cellular and animal studies, with relatively few well-designed clinical trials. Correspondingly, pharmacodynamic, pharmacokinetic, and safety data remain inadequate to support robust clinical translation. Finally, systematic investigations of combination regimens involving saponins and established therapies for HUA and gout are still limited, particularly with respect to their potential synergistic efficacy and safety.

Future research should therefore place greater emphasis on identifying the core pharmacologically active constituents of saponin preparations, defining their molecular targets, and establishing clear dose–effect and dose–toxicity relationships, thereby providing a stronger theoretical and experimental basis for the development of saponin-based therapeutics. Modern formulation and drug-delivery technologies should also be exploited to improve oral bioavailability and enhance targeted delivery. At the clinical level, standardized and adequately designed trials are needed to systematically evaluate therapeutic efficacy and safety and to facilitate the translation of preclinical findings into clinical practice. In parallel, combination strategies incorporating saponins derived from Chinese medicinal materials with first-line therapies for HUA and gout warrant further investigation to determine whether synergistic benefits can be achieved without compromising safety. Collectively, these efforts may accelerate the clinical translation of saponin-based interventions and provide safer and more effective therapeutic options for patients with HUA and gout.

## Figures and Tables

**Table 1:** Characteristics of animal models used for evaluating the hypouricemic and anti-gout effects of saponin compounds.

Model	Modeling Method	SUA Change	Main Characteristics	Ref.
PO-induced HUA	PO administration (rats: 250 mg/kg/d, 7 days; mice: 250 mg/kg/d, 21 days)	Rats: approximately 1.5–2.1-fold increase; Mice: approximately 1.87-fold increase	Rapid and reproducible model widely used for evaluating urate-lowering activity	[66]
PO- and hypoxanthine-induced HUA	PO injection (100 mg/kg/d) combined with hypoxanthine gavage (500 mg/kg/d) for 7 days	Mice: Increased from 184.65 ± 4.81 to 430.19 ± 36.31 μmol/L	Produces a relatively stable hyperuricemic state	[67]
Adenine- and ethambutol-induced HUA	Adenine (200 mg/kg) and ethambutol (250 mg/kg) gavage for 28 days	Rats: SUA increased approximately 2.5-fold (control: 110 μmol/L)	Mimics hyperuricemia associated with impaired urate excretion	[68]
Yeast extract-induced HUA	High-purine yeast extract gavage (30 g/kg, twice daily) for 8 days	Mice: SUA increased from 77 ± 11 to 118 ± 23 μmol/L (approximately 53% increase)	Mimics hyperuricemia associated with high-purine dietary intake	[69]
PO- and yeast diet-induced HUA	PO (750 mg/kg/d) combined with a 20% high-yeast diet for 21 days	SUA levels increased from 88.0 ± 14.1 μmol/L to 254.6 ± 23.7 μmol/L	Provides a relatively stable hyperuricemic state	[70]
PO-and fructose-induced HUA	PO (100 mg/kg) combined with fructose (5% or 10%)	Mice: SUA levels were significantly increased and accompanied by metabolic abnormalities	Mimics hyperuricemia associated with high fructose intake	[71]
Lipid emulsion-induced HUA	Lipid emulsion gavage (10 mL/kg/d) for 8 weeks	Rat: SUA levels were significantly increased compared with controls	Mimics hyperuricemia associated with high-fat and high-cholesterol metabolic disturbances	[72]
MSU crystal-induced GA	Intra-articular injection of MSU crystals	SUA levels generally remain unchanged	Mainly used for evaluating anti-inflammatory and anti-gout effects	[73]

**Table 2:** Comparative Pharmacological Profiles of CMM-Derived Saponins in Hyperuricemia and Gout.

Mechanism	Saponin Components	Experimental Model	Dose/Duration	Mechanism	Quantitative Change	Refs.
Purine-metabolizing enzymes	Total saponins from *S. riparia*	PO-induced HUA mice	500 mg/kg; 7 days	XOD ↓ ≈ 23%	SUA ↓ ≈ 19%	[74]
	Pallidifloside D	PO-induced HUA mice	5 mg/kg; 7 days	XOD ↓ ≈ 2%, PRPS, PRPPAT mRNA ↓; HGPRT mRNA ↑	SUA ↓ ≈ 11%;	[75,76]
	Smilaxchinoside A	PO-induced HUA mice	10 mg/kg; 7 days	XOD ↓ ≈ 27%	SUA ↓ ≈ 22%	[77]
	Smilaxchinoside C	PO-induced HUA mice	10 mg/kg; 7 days	XOD ↓ ≈ 25%	SUA ↓ ≈ 21%	[77]
	Riparoside B	PO-induced HUA mice	10 mg/kg; 7 days	XOD ↓ ≈ 22%	SUA ↓ ≈ 23%	[78]
	Timosaponin J	PO-induced HUA mice	10 mg/kg; 7 days	XOD ↓ ≈ 19%	SUA ↓ ≈ 20%	[78]
	Gypenosides	Lipid emulsion-induced HUA rats	60 mg/kg; 8 weeks	XOD ↓ = 18%, ADA ↓ = 10%, XDH = ↓ 22%, GDA ↓ 11%	SUA ↓ = 32.7%	[79]
	Saikosaponin D	Adenine- and ethambutol-induced HUA rats	100, 200 mg/kg; 28 days	XOD ↓ ≈ 19–22% ADA ↓ ≈ 48–62%,	SUA ↓ ≈ 22–28%	[80]
	Total saponins from *Dioscorea nipponica* Makino	PO-induced HUA mice	600 mg/kg; 7 days	XOD ↓ ≈ 15%, ADA ↓ ≈14%	SUA ↓ = 29–43%	[81]
	Total saponins from *Apostichopus japonicus*	Yeast powder-induced HUA mice	0.05, 0.1% in diet; 14 days	XOD ↓ = 26.3%, 28.6% ADA ↓ = 24.4%, 34.0%	SUA ↓ = 53.1, 55.6%	[82]
	Flaccidoside II	PO-induced HUA mice	8, 16, 32 mg/kg; 7 days	32 mg/kg: XOD ↓ ≈ 15%	SUA ↓ = 26.1–30.9%	[83]
Urate transporters	Total saponins from *Smilax riparia* A. DC.	PO-induced HUA mice	500 mg/kg, 7 days	URAT1 ↓ ≈ 12% and GLUT9 ↓ ≈ 5%; OAT1 ↑ ≈ 32%	SUA ↓ ≈19%	[74]
	Pallidifloside D	PO-induced HUA mice	5 mg/kg; 7 days	URAT1 ↓ ≈7% and GLUT9 ↓ ≈ 6%; OAT1 ↑ ≈ 14%	SUA ↓ ≈ 11%	[76]
	Smilaxchinoside A	PO-induced HUA mice	10 mg/kg, 7 days	URAT1 ↓ ≈ 13%	SUA ↓ ≈ 22%	[77]
	Smilaxchinoside C	PO-induced HUA mice	10 mg/kg, 7 days	URAT1 ↓ ≈ 12%	SUA ↓ ≈ 21%	[77]
	Riparoside B	PO-induced HUA mice	10 mg/kg; 7 days	URAT1 ↓ ≈ 18%	SUA ↓ ≈ 23%	[78]
	Timosaponin J	PO-induced HUA mice	10 mg/kg, 7 days	URAT1 ↓ ≈ 10%	SUA ↓ ≈ 20%	[78]
	Gypenosides	Lipid emulsion-induced HUA rats	60 mg/kg; 8 weeks	URAT1 ↓ ≈ 48% and GLUT9 ↓ ≈ 68%; OAT ↑ ≈ 124%	SUA ↓ = 32.7%	[79]
	Total saponins from *Dioscorea nipponica* Makino	Adenine- and ethambutol-induced HUA rats, PO-induced HUA mice	40 mg/kg, 7 days	URAT1 ↓ ≈ 35%; OAT1 ↑ ≈ 44%; OAT3 ↑ ≈ 33%; ABCG2 ↑ ≈ 63%	SUA ↓ ≈ 22%	[84,86]
	Total saponins from *Dioscorea collettii* Hook. f.	Adenine- and ethambutol-induced HUA rats	300 mg/kg	URAT1 ↓ ≈ 42%; GLUT9 ↓ ≈ 33%; OAT1 ↑ ≈ 31%; OAT3 ↑ ≈ 25%	SUA ↓ ≈ 22%	[85]
	Saikosaponin A	Uric acid-injured HK-2 cells	10 μM	URAT1 mRNA ↓ ≈ 70%; GLUT9 mRNA ↓ ≈ 57%; ABCG2 mRNA ↑ ≈ 322%	-	[87]
	Crude saponin extract from leaves of *Ilex cornuta* Lindl. & Paxton	PO- and yeast powder-induced HUA mice	50, 100 mg/kg; 7 days	URAT1 ↓ ≈ 53%; GLUT9 ↓ ≈ 67%; ABCG2 ↑ ≈ 162%	SUA ↓ ≈ 22–26%	[88]
	Total saponins from stems and leaves of *Achyranthes bidentata* Bl.	Adenine- and ethambutol-induced HUA rats	100, 200 mg/kg, 28 days	URAT1 ↓ ≈ 10–25%; GLUT9 ↓ ≈ 5–20%; OAT1 ↑ ≈ 10–25%	SUA ↓ ≈ 20–30%	[89]
	Total saponins from *Clematis chinensis* Osbeck	PO- and hypoxanthine-induced HUA mice	50 mg/kg; 7 days	URAT1 mRNA ↓ ≈ 50%; GLUT9 mRNA ↓ ≈ 50%; OAT3 ↑ ≈ 104%; ABCG2 ↑ ≈ 43%	SUA ↓ ≈ 65%	[90]
	Clematichinenoside AR	PO- and hypoxanthine-induced HUA mice	12.5, 25, 50 mg/kg; 7 days	OAT1 ↑ ≈ 190%; OAT3 ↑ ≈ 160%; ABCG2 ↑ ≈ 169%	SUA ↓ ≈ 72–85%	[91]
Gut microbiota	Astragalus saponins	PO- and fructose-induced HUA mice	1.5 g/kg; 24 days (twice daily)	*Lachnospiraceae*, *Muribaculaceae*, and *Clostridia* ↑; F/B ratio and *Escherichia–Shigella* ↓; XOD ↓	SUA ↓ ≈ 30%	[92,93]
	Ginsenoside Rg1	PO plus a yeast extract-containing high-purine diet-induced HUA rats	12.5, 25 mg/kg, 8 weeks	*Lactobacillus*, *Lactococcus*, *Ruminococcus* and *Lachnospiraceae_NK4A136_group* ↑; occludin and ZO-1 ↑, XOD ↓	SUA ↓ ≈ 49–55% SCFAs ↑ ≈ 29–45%	[94]
	Rare ginsenosides (Rg3S, Rg3R, F4, Rg6, Rk1,Rg5)	PO-induced HUA mice	50, 100, 200 mg/kg, 35 days	F/B ratio ↓; *Lactobacillus* and *Akkermansia* ↑	SUA ↓ ≈ 25–42%	[95]
	Total saponins from *Dioscorea nipponica* Makino	PO-induced HUA mice; MSU-induced GA rats	40, 200, 400 mg/kg, 14 days	*Firmicutes*, *Campylobacterota*, *Lactobacillus*, *Clostridium* and *Ruminococcus* ↑; *Bacteroidota*, *Bacteroides* and *Parabacteroides* ↓;	SUA ↓ ≈ 34–62%	[96,97]
	Total saponins from *Brassica rapa* L.	PO- and hypoxanthine-induced HUA mice	500, 1000, 1500 mg/kg, 14 days	*Paramicrobium*, *Duncaniella, Duncaniella* and *Eubacterium_F* ↑; *CAG-873* and *CAG-485* ↓; intestinal ABCG2 ↑	SUA ↓ ≈ 15–50%; acetate ↓ = 16.9%; butyrate ↑ = 41.2%	[98]
Inflammatory signaling pathways	Total saponins from *Dioscorea nipponica* Makino	MSU crystal-induced GA rats	160 mg/kg	TLR2/4, IRAK1, TRAF6, TAK1, IKKα, IκBα, and NF-κB p65 ↓; MEK1/2, ERK1/2, MKK4, JNK, ICAM-1, and CXCL1 ↓; caspase-1, ASC, TNF-α, IL-1β, and IL-6 ↓	IL-1β ↓ ≈ 33%; TNF-α mRNA ↓ ≈ 27%	[99,100,101]
	Dioscin	MSU crystal-induced GA mice and rats	50, 100 mg/kg; 7 days	IKKβ,NF-κB p65 ↓;NLRP3, ASC, Caspase-1,IL-1β ↓; MKK3/6 ↓	IL-1β ↓ ≈ 56%, 65%; IL-6 ↓ ≈ 23%, 66%; TNF-α ↓ ≈ 67%, 80%	[102,103,104]
	Ginsenoside Rb1	MSU crystal-induced GA rats	80 mg/kg; 9 days	IκBα degradation ↓; mitochondrial damage alleviated	IL-1β/IL-6 mRNA ↓ ≈ 47%; TNF-α mRNA ↓ ≈ 41%	[105]
	Ginsenoside Rg1	MSU crystal-induced GA rats	12.5, 25 mg/kg; 7 days	TLR4, MyD88, NLRP3, ASC, and caspase-1 ↓; p38, ERK, and JNK phosphorylation ↓; NETs formation ↓	IL-1β ↓ ≈ 21%, 17%; IL-6 ↓ ≈ 34%, 27%	[106]
	Madecassoside	MSU crystal-induced GA rats	10, 20, 40 mg/kg; 2 weeks	NF-κB, NLRP3, ASC, and caspase-1 ↓; NLRP3 inflammasome activation ↓	IL-1β ↓ ≈ 25%; TNF-α ↓ ≈ 8%	[107,108]
	Anemoside B4	PO-induced HUA rats; MSU-induced GA rats	5, 10, 20 mg/kg; 7 days	IKKα/β, IκBα, and NF-κB p65 phosphorylation ↓; mitochondrial damage alleviated; ROS ↓	IL-1β ↓ ≈ 12–14%; IL-6 ↓ ≈ 13–30%; TNF-α ↓ ≈ 6–8%	[109]
Inflammatory signaling pathways	Total saponins from *Dioscorea hypoglauca* Palibin	MSU crystal-induced THP-1 cell model of GA and GA rat model	30, 100, 300 mg/kg; 7 days	TLR4, NF-κB, and NF-κB p65 ↓; NLRP3, ASC, and pro-caspase-1 ↓	IL-1β ↓ ≈ 12–35%; TNF-α ↓ ≈ 7–45%	[110,111]
	Total saponins from *Panax notoginseng* (Burk.) F.H. Chen	MSU-induced GA mouse model; HUVEC model	35, 70, 140 mg/kg; 5 days	NLRP3, ASC, and caspase-1 ↓; NLRP3 inflammasome activation ↓; IL-1β, IL-18, TNF-α, and ICAM-1 ↓	IL-1β ↓ ≈ 29%; TNF-α ↓ ≈ 13%	[112,115]
	Total saponins from *Platycodon grandiflorus* (Jacq.) A. DC.	MSU crystal-induced GA rats	25, 50, 100 mg/kg; 7 days	NLRP3, ASC, and caspase-1 ↓; IL-6, IL-1β, and TNF-α ↓	TNF-α ↓ ≈ 18–27%	[100]
	Clematichinenoside AR	PO- and hypoxanthine-induced HUA mice	12.5, 25, 50 mg/kg; 7 days	NLRP3 and caspase-1 ↓	IL-1β ↓ ≈ 45–53%; IL-6 ↓ ≈ 14–46%; TNF-α ↓ ≈ 58–63%	[91]
	Doliroside A	MSU crystal-induced GA rats	10, 20, 40 mg/kg; 4 days	caspase-1 and pro-IL-1β ↓; NLRP3 inflammasome activation ↓	IL-1β ↓ ≈ 21–55% TNF-α ↓ ≈ 18–41%	[114]
	Total saponins from *Smilax nipponica* Miq.	MSU-induced GA rats	85, 340 mg/kg	PI3K and Akt ↓	IL-1β ↓ ≈ 30%, 36%; IL-6 ↓ ≈ 28%, 35%; TNF-α ↓ ≈ 9%, 19%	[116]
	Smilnipponicosides A, Smilnipponicosides C	MSU-stimulated RAW264.7 cell model	60, 120 μM	PI3K, p-PI3K, and p-Akt ↓	-	[116]

Note: Values are approximated from the data reported in the original studies. SUA reduction percentages were calculated relative to the HUA model group unless otherwise indicated. “-” indicates that the parameter was not reported or not applicable. ↑ indicates an increase; ↓ indicates a decrease.

**Table 3:** Structural Characteristics of Representative Saponins from CMMS and Their Associations with Hypouricemic and Anti-gout Activities.

Saponin	Saponin Class/ Aglycone Skeleton	Aglycone Structure	Sugar Chain Composition and Linkage Pattern	Refs.
Pallidifloside D	Pregnane-type C22 steroidal lactone saponin	3β,16β-dihydroxy-pregn-5,20-dien-carboxylic acid (22,16)-lactone	C-3: α-L-Rha-(1 → 2)-β-D-Glc	[76]
Smilaxchinoside A	Furostan-type steroidal saponins; 25S configuration	3β,20α,26-trihydroxyfurosta-5,22-diene	C-26: β-D-Glc; C-3: α-L-Rha-(1 → 2)-[α-L-Rha-(1 → 4)]-β-D-Glc	[77]
Smilaxchinoside C	Furostan-type steroidal saponins; 25R configuration	3β,20α,26-trihydroxyfurosta-5,22-diene	C-26: β-D-Glc; C-3: α-L-Rha-(1 → 2)-β-D-Glc	[77]
Riparoside B	Furostan-type Steroid Saponins	3,20-Dihydroxy-furost-22(23)-ene aglycone	C-26: β-D-Glc; C-3: α-L-Rha-(1 → 2)-[α-L-Rha-(1 → 6)]-β-D-Glc	[78]
Timosaponin J	20,22-secofurostane-type steroidal saponins	3-Hydroxy-20,22-secofurostane-20,22-dione aglycone	C-26: β-D-Glc; C-3: β-D-Glc-(1 → 2)-β-D-Gal	[78]
Saikosaponin D	Oleanane-type pentacyclic triterpenoid saponin	Saikogenin G	C-3: β-D-Fuc-(1 → 2)-β-D-Glc	[117]
Flaccidoside II	Oleanane-type pentacyclic triterpenoid saponin	Oleanolic acid	C-3: α-L-Rha-(1 → 2)-β-D-Xyl; C-28: α-L-Rha-(1 → 4)-β-D-Glc-(1 → 6)-β-D-Glc	[83]
Saikosaponin A	Oleanane-type pentacyclic triterpenoid saponin	Saikogenin F	C-3: β-D-Fuc-(1 → 2)-β-D-Glc	[117]
Clematichinenoside AR	Oleanane-type pentacyclic triterpenoid saponin	Oleanolic acid	C-3: α-L-Rha-(1 → 6)-β-D-Glc-(1 → 4)-β-D-Glc-(1 → 4)-β-D-Rib-(1 → 3)-α-L-Rha-(1 → 2)-α-L-Ara; C-28: α-L-Rha-(1 → 4)-β-D-Glc-(1 → 6)-β-D-Glc	[118]
Ginsenoside Rg1	Dammarane-type triterpenoid saponin; protopanaxatriol (PPT)-type	20(S)-Protopanaxatriol	C-6: β-D-Glc; C-20: β-D-Glc	[119]
Rare ginsenosides (Rg3S, Rg3R, F4, Rg6, Rk1, Rg5)	Low-glycosylated rare ginsenosides; protopanaxadiol (PPD)/protopanaxatrio-type (PPD)dammarane skeletons	Rg3(S)/Rg3(R), Rk1, and Rg5: PPD-type; F4 and Rg6: PPT-type, accompanied by C-20 configuration changes or side-chain dehydration/unsaturation	Rg3(S)/Rg3(R), Rk1, and Rg5 mainly retain the C-3-linked Glc-(1 → 2)-Glc disaccharide chain, whereas F4 and Rg6 mainly contain the C-6-linked Rha-(1 → 2)-Glc disaccharide chain	[95]
Dioscin	Spirostane-type steroidal saponin	Diosgenin	C-3: α-L-Rha-(1 → 2)-[α-L-Rha-(1 → 4)]-β-D-Glc	[104]
Ginsenoside Rb1	Dammarane-type triterpenoid saponin; PPD-type	20(S)-Protopanaxadiol	C-3: β-D-Glc-(1 → 2)-β-D-Glc; C-20: β-D-Glc-(1 → 6)-β-D-Glc	[119]
Madecassoside	Ursane-type pentacyclic triterpenoid saponin	Madecassic acid	C-28: ester-linked trisaccharide chain: α-L-Rha-(1 → 4)-β-D-Glc-(1 → 6)-β-D-Glc	[120]
Anemoside B4	Lupane-type pentacyclic triterpenoid saponin	23-Hydroxybetulinic acid	C-3: α-L-Rha-(1 → 2)-α-L-Ara; C-28: α-L-Rha-(1 → 4)-β-D-Glc-(1 → 6)-β-D-Glc	[121]
Doliroside A	Oleanane-type pentacyclic triterpenoid saponin	Medicagenic acid 23-monomethylate	C-3: β-D-Glc(3-O-β-D-glucopyranoside)	[114]
Smilnipponicoside A	Pregnane-type steroidal glycoside	Pregna-16-en-20-one steroidal aglycone	C-3: β-D-Glc-(1 → 4)-[α-L-Rha-(1 → 6)]-β-D-Glc	[122]
Smilnipponicoside C	Pregnane-type steroidal glycoside	Pregna-5,16-dien-20-one steroidal aglycone	C-3: β-D-Glc-(1 → 2)-[α-L-Rha-(1 → 6)]-β-D-Glc	[122]

**Table 4:** Oral pharmacokinetic characteristics and bioavailability of representative individual saponins.

Saponin	Species	Dose (mg/kg)	C_max_ (ng/mL)	T_max_ (h)	t_1/2_ (h)	AUC (ng·h/mL)	F (%)	Ref.
Saikosaponin D	Rat	200	2730 ± 350	1.6 ± 0.22	1.25 ± 0.13	AUC_0–∞_: 9570 ± 1030	NR	[125]
Saikosaponin A	Rat	50	23.43 ± 7.79	1.63 ± 0.25	5.65 ± 1.74	AUC_0–∞_: 4.03 ± 1.19	0.04	[126]
		100	41.73 ± 17.97	1.75 ± 0.29	7.11 ± 1.27	AUC_0–∞_: 7.57 ± 3.42		
		200	79.84 ± 51.63	2.00 ± 0.71	6.82 ± 1.27	AUC_0–∞_: 14.68 ± 9.40		
Clematichinenoside AR	Rat	8	59.73 ± 25.69	3.83 ± 2.07	4.10 ± 2.36	AUC_0–∞_: 358.17 ± 135.88	NR	[127]
		16	76.30 ± 22.71	2.50 ± 0.84	4.30 ± 2.06	AUC_0–∞_: 684.06 ± 383.33		
		32	197.57 ± 61.81	1.83 ± 0.75	3.50 ± 1.94	AUC_0–∞_: 1041.57 ± 322.78		
Ginsenoside Rg1	Rat	150	1134.4 ± 562.8	0.92 ± 0.12	2.25 ± 0.68	AUC_0–T_: 2363.5 ± 914.8	NR	[128]
Ginsenoside Rb1	Rat	50	6100 ± 3400	2.0 ± 0.9	9.8 ± 6.9	AUC_0–∞_: 66,800 ± 34,000	0.78	[129]
Dioscin	Rat	45	9.30 ± 0.91	19.4 ± 0.98	15.2 ± 2.19	AUC_0–∞_: 536 ± 78.0	0.23	[130]
		90	16.5 ± 1.48	16.1 ± 3.54	26.6 ± 2.36	AUC_0–∞_: 1002 ± 46.0	0.21	
Madecassoside	Rat	30	27.144 ± 9.766	1.321 ± 0.874	5.007 ± 0.299	AUC_0–∞_: 243.112 ± 83.687	NR	[131]
Anemoside B4	Rat	122	94.47 ± 28.99	2.33 ± 1.53	2.93 ± 1.04	AUC_0–∞_: 592.55 ± 57.78	<1	[132]

Notes: Values are reported as presented in the original studies. F, absolute oral bioavailability; NR, not reported; C_max_, maximum plasma concentration; T_max_, time to reach C_max_; t_1/2_, terminal half-life; AUC, area under the plasma.

**Table 5:** Toxicological findings of representative saponins and total saponin extracts.

Saponin Components	Model and Exposure	Key Toxicological Findings	Ref.
Total saponins from *Dioscorea nipponica* Makino	Rats; oral administration of 50, 150, and 450 mg/kg/day for 28 days	Increased ALT and mild reversible centrilobular hydropic changes	[104]
Total saponins from *Panax notoginseng* (Burk.) F.H. Chen	rats; oral administration 1.6 g/kg, 6 d/week for 13 weeks	At 1.6 g/kg, increased AST/ALT and liver weight, with hepatocellular vacuolar degeneration; biochemical changes were reversible	[134]
Saikosaponin A	Mice; oral administration of 75, 150, and 300 mg/kg/day for 5 days	At 150–300 mg/kg/day, increased liver/body-weight ratio and hepatic histological injury; ER stress/autophagy dysregulation	[135]
Saikosaponin D	LO2 hepatocytes; 0.4–2 μM	Concentration-dependent hepatotoxicity (IC_50_ ≈ 2.14 μM); apoptosis at higher exposure	[136]
Dioscin	LO2 hepatocytes; rats, oral administration up to 300 mg/kg/day for 90 days	Hepatocyte toxicity in vitro; gastrointestinal and mild hematological changes after prolonged high-dose exposure	[137]
Flaccidoside II	Mice; single oral administration of 300 mg/kg; 14-day observation	No mortality or overt acute toxicity	[83]
Anemoside B4	HEK293 cells; mice, intraperitoneal administration of 2.5 g/kg/day for 14 days	No significant changes in body weight, ALT, AST, BUN, or creatinine	[138]
Madecassoside	HaCaT cells and zebrafish models	No significant HaCaT cytotoxicity at ≤1000 μg/mL; growth inhibition at high concentrations in zebrafish	[139,140]
Ginsenoside Rg1	Rat and mouse whole-embryo culture; 10, 30, and 50 μg/mL, 48 h	Impaired embryonic growth from 10 μg/mL; morphological abnormalities at higher concentrations	[141]
Ginsenoside Rb1	Mouse blastocysts; 25–100 μg/mL for 24 h	At 50–100 μg/mL, increased apoptosis and reduced ICM/TE cell numbers; impaired embryonic development	[142]

## Data Availability

No new data were created or analyzed in this study. Data sharing is not applicable to this article.

## Abbreviations

The following abbreviations are used in this manuscript:

HUA	Hyperuricemia
SUA	Serum uric acid
MSU	Monosodium urate
TCM	Traditional Chinese Medicine
CMMs	Chinese medicinal materials
XOD	Xanthine oxidase
ADA	Adenosine deaminase
HGPRT	Hypoxanthine–guanine phosphoribosyltransferase
PRPP	Phosphoribosyl pyrophosphate
URAT1	Urate transporter 1
GLUT9	Glucose transporter 9
ABCG2	ATP-binding cassette subfamily G member 2
OAT1	Organic anion transporter 1
OAT3	Organic anion transporter 3
NF-κB	Nuclear factor-κB
NLRP3	NLR family pyrin domain-containing 3
MAPK	Mitogen-activated protein kinase
PI3K	Phosphoinositide 3-kinase
AKT	Protein kinase B
ROS	Reactive oxygen species
LPS	Lipopolysaccharide
TLR4	Toll-like receptor 4
MyD88	Myeloid differentiation primary response 88
TNF-α	Tumor necrosis factor-α
IL-1β	Interleukin-1β
IL-6	Interleukin-6
ASC	Apoptosis-associated speck-like protein containing a caspase recruitment domain
SCFAs	Short-chain fatty acids
F/B	*Firmicutes*/*Bacteroidetes*
ZO-1	Zonula occludens-1
